# Biomaterial Surface‐Mediated Macrophages Exert Immunomodulatory Roles by Exosomal CCL2‐Induced Membrane Integrin β1 Trafficking in Recipient Cells

**DOI:** 10.1002/advs.202409809

**Published:** 2025-01-21

**Authors:** Yuyu Zhao, Ruiyue Hang, Huifei Li, Yonghua Sun, Runhua Yao, Xiaobo Huang, Xiangyu Zhang, Xiaohong Yao, Huaiyu Wang, Yin Xiao, Di Huang, Yong Han, Xing Wang, Ruiqiang Hang

**Affiliations:** ^1^ Shanxi Key Laboratory of Biomedical Metal Materials College of Materials Science and Engineering Taiyuan University of Technology Taiyuan 030024 China; ^2^ School and Hospital of Stomatology Shanxi Medical University Taiyuan 030001 China; ^3^ Center for Human Tissues and Organs Degeneration Shenzhen Institute of Advanced Technology Chinese Academy of Sciences Shenzhen 518055 China; ^4^ School of Medicine and Dentistry Griffith University Gold Coast QLD 4222 Australia; ^5^ Research Center for Nano‐Biomaterials & Regenerative Medicine Department of Biomedical Engineering College of Biomedical Engineering Taiyuan University of Technology Taiyuan 030024 China; ^6^ State‐Key Laboratory for Mechanical Behavior of Materials Xi'an Jiaotong University Xi'an 710049 China

**Keywords:** biomaterials, exosomes, immunomodulation, macrophages, tissue regeneration

## Abstract

The interaction between biomaterials and immune system is a critical area of research, especially in tissue engineering and regenerative medicine. A fascinating and less explored aspect involves the immunomodulatory behaviors of macrophage (MΦ)‐derived exosomes induced by biomaterial surfaces. Herein, untreated surface, nanostructured surface, and type I collagen (Col‐I)‐decorated nanostructured surface of titanium implants are chosen to culture MΦs, followed by extraction of MΦ‐derived exosomes and investigation of their immunomodulatory functions and mechanisms. The results show that the exosomes in the untreated group carried plenty of inflammatory cytokines, predominantly C*─*C motif chemokine ligand 2 (CCL2). After targeting recipient cells, the CCL2 on the exosomes can specifically bind to its receptor C*─*C motif chemokine receptor 2, triggering downstream signaling pathways to induce internalization of membrane integrin β1 and targeted lysosomal degradation, consequently suppressing the functions of recipient cells. In contrast, the exosomes in the nanostructured group, especially Col‐I‐decorated nanostructured group carried few CCL2, moderating their inhibition on the functions of recipient cells. These findings not only clearly show that CCL2 is a key constituent of exosomes involved in the interaction between biomaterials and host immune system, but also potentially a key target for designing advanced biomaterials to promote tissue repair and regeneration.

## Graphical abstract

Biomaterial‐mediated macrophage‐derived exosomes inhibit the spreading and function of tissue repair cells by inducing internalization and degradation of recipient cell membrane integrin β1 via their outer membrane‐bound inflammatory cytokines (predominantly C─C motif chemokine ligand 2 (CCL2)). Appropriate surface characteristics of biomaterials can reduce the amounts of inflammatory cytokines bound to the macrophage‐derived exosomes, thereby attenuating their inhibition of recipient cell function.

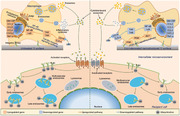

## 1 Introduction

As a pillar of regenerative medicine, biomaterials can mediate endogenous tissue repair and regeneration without the need to deliver cells or other therapeutics.^[^ ^1^, ^2^ ^]^ Once biomaterials come into contact with host tissues, they are bound to elicit host innate immune responses and inflammation, which were once thought to be harmful forces that needed to be attenuated or avoided.^[^ ^3^ ^]^ However, recent studies showed if properly designed, the biomaterials could create a favorable immune microenvironment to promote tissue repair and regeneration,^[^ ^4^, ^5^, ^6^, ^7^ ^]^ which spawns the immunomodulation‐based strategies for the design of advanced biomaterials.^[^ ^3^, ^8^, ^9^ ^]^ Macrophages (MΦs) are essential components of the immune system and act as major effectors of innate immune responses, exhibiting remarkable phenotypic and functional plasticity.^[^ ^10^, ^11^, ^12^ ^]^ Generally, MΦs can be activated to proinflammatory M1 phenotype (classically activated phenotype) to secret multiple inflammatory cytokines (e.g., interleukin‐1β (IL‐1β), IL‐6, MΦ colony‐stimulating factor (M‐CSF), and tumor necrosis factor‐α (TNF‐α)) as inhibitors of tissue repair and regeneration. Conversely, MΦs polarized to anti‐inflammatory M2 phenotype (alternatively activated phenotype) can secrete various growth factors, such as vascular endothelial growth factor (VEGF), bone morphogenetic protein 2 (BMP2), platelet‐derived growth factor (PDGF), and transforming growth factor (TGF‐β). Mediating MΦ polarization through rational biomaterial design has emerged as key to promoting tissue repair and regeneration.^[^ ^12^, ^13^ ^]^

Exosomes, bilayer lipid small extracellular vesicles that are actively secreted by mammalian cells, have garnered increasing attention in the field of tissue engineering and regenerative medicine.^[^ ^2^, ^14^ ^]^ Exosomes exert a crucial role in intercellular communication, both locally and distally. They can transport proteins, lipids, and nucleic acids to recipient cells, thereby affecting their functions.^[^ ^15^, ^16^, ^17^ ^]^ Cumulative pieces of evidences reveal that MΦ‐derived exosomes show prominent abilities in regulating tissue repair and regeneration. The exosomes from M1 MΦs transferred proinflammatory miRNAs (such as miR‐155) to endothelial cells (ECs), thus inhibiting angiogenesis.^[^ ^18^ ^]^ On the contrary, M2 MΦ‐derived exosomes promoted the recovery of spinal cord injuries through stimulating vascular regeneration.^[^ ^19^ ^]^ Furthermore, M2 MΦ‐derived exosomes transmitted mRNA and miRNA to guide gene expression in bone marrow mesenchymal stem cells (BMSCs), enhancing osteogenic differentiation and bone tissue regeneration.^[^ ^20^, ^21^ ^]^ Contrarily, exosomes derived from M1 MΦs suppressed osteogenic‐related gene transcription and osteogenesis of BMSCs. The incorporation of M2 MΦ‐derived exosomes substantially improved the proangiogenic and pro‐osteogenic activities of biomaterials.^[^ ^22^, ^23^, ^24^ ^]^ Collectively, the exosomes derived from MΦs with different phenotypes play distinct regulatory roles. It is well known that biomaterial characteristics can effectively mediate the phenotype polarization of MΦs, which in turn regulate the functions of cells involved in tissue repair and regeneration. Nonetheless, the roles and mechanisms of MΦ‐derived exosomes mediated by biomaterials on tissue repair and regeneration are less explored but critically important for the rational design of advanced biomaterials.

**Figure 1:**
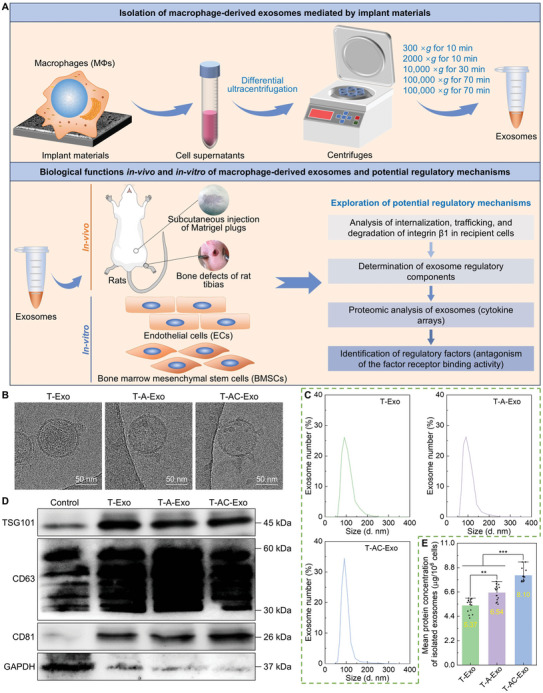
Research processes and characterization of the MΦ‐derived exosomes. A) Schematic diagram of the research processes. B) Representative Cryo‐TEM micrographs of the exosomes. C) Size distribution of the exosomes detected by DLS. D) Western blotting analysis for the exosome‐specific markers. E) Protein concentrations of the isolated exosomes (*n* = 15). T‐Exo: exosomes derived from the MΦs cultured on untreated pure Ti surface (T), T‐A‐Exo: exosomes derived from the MΦs cultured on nanostructured Ti surface (T‐A), and T‐AC‐Exo: exosomes derived from the MΦs cultured on Col‐I‐decorated nanostructured Ti surface (T‐AC). Data are presented as means ± SD. ^**^ *p* < 0.01 and ^***^ *p* < 0.001.

In the case of implantable biomaterials, two critical cues, topography and biochemical composition, determine the immune responses of MΦs. Our previous study has suggested that nanoporous structure and type I collagen (Col‐I) decoration exhibited significantly differential modulation on titanium (Ti) implant‐mediated MΦ immune responses and phenotype polarization.^[^ ^4^ ^]^ In this work, nanoporous structure and Col‐I were chosen to modify the surface of Ti substrates to investigate the effects of implant surface physicochemical characteristics on immunomodulatory functions and mechanisms of MΦ‐derived exosomes. Herein, the exosomes derived from MΦs cultured on three groups of specimens (untreated pure Ti (T), nanostructured Ti (T‐A), and Col‐I‐decorated nanostructured Ti (T‐AC)) were successfully isolated, and the effects of the exosomes on the cellular behaviors and functions of ECs and BMSCs and the potential regulatory mechanisms were investigated (**Figure** **1**A). We firmly believe the knowledge gained from the present work can enrich the theory of biomaterial‐mediated osteoimmunology and direct the design of immunomodulation‐based biomaterials.

## 2 Results

### 2.1 Extraction and Characterization of MΦ‐Derived Exosomes

As shown in Figure S1 (Supporting Information), the untreated surface T showed a flat topography, and the alkali‐treated surface T‐A presented a nanoporous structure. The Col‐I‐decorated nanostructured surface T‐AC still possessed the nanoporous structure and exhibited obvious chemical characteristics of Col‐I (peaks of C═O, N*─*H, and C*─*H and organic constituent chemical elements), with the surface‐immobilized Col‐I of about 73.86 µg cm^−2^. Three sets of the specimens supported the viability (intense green fluorescence) and proliferation of lipopolysaccharide (LPS)‐activated MΦs (Figure S2A,C, Supporting Information). Cell morphology of the MΦs cultured on T and T‐A appeared bulging sphere‐shape, whereas the MΦs cultured on T‐AC exhibited a flat shuttle‐shape with outspread lamellipodia at the edge of the cells and abundant filopodia on the cell surfaces (Figure S2B,D, Supporting Information). Previous study has demonstrated that the sphere‐shaped MΦs exhibited a trend toward the M1 phenotype and shuttle‐shape MΦs exhibited a trend toward the M2 phenotype.^[^ ^4^ ^]^ This tendency was also confirmed by immunofluorescence staining of M1/M2 phenotypic markers (Figure S2E, Supporting Information). The control group was LPS‐activated MΦs which exhibited a typical M1‐polarized phenotype. The MΦs cultured on T showed a higher level of inducible nitric oxide synthase (iNOS), indicating a potent tendency toward M1‐phenotypic polarization. T‐A significantly downregulated iNOS level and upregulated MΦ mannose receptor C‐type 1 (CD206) level in MΦs compared with T, suggesting that the nanoporous structure potentially inhibited MΦ polarization toward the M1 phenotype and promoted it toward the M2 phenotype. T‐AC mediated the lowest iNOS level and the highest CD206 level, demonstrating that the Col‐I‐decorated nanostructured surface effectively modulated MΦs polarizing to the M2 phenotype.

Supernatants derived from MΦs cultured on the specimen surfaces were collected and used to isolate exosomes. The cryo‐transmission electron microscope (Cryo‐TEM) micrographs demonstrated that the exosomes were round‐like vesicles with a bilayer lipid membrane and there was no difference in the appearance of three groups of the exosomes (Figure 1B). The field‐emission scanning electron microscopy (FE‐SEM) images showed that the exosomes in spherical nanoparticle‐like morphology were uniformly distributed in the field of view (Figure S3A, Supporting Information). The histogram of the statistical analysis for nanoparticle size distribution in the FE‐SEM images revealed that the size of the isolated exosomes was concentrated in the range of 40–150 nm, which is the typical size of exosomes (Figure S3C, Supporting Information). In addition, three groups of the exosomes exhibited a predominant size of 80–110 nm. Exosome size was verified by dynamic light scattering (DSL) and all exosomes were enriched around 100 nm in diameter (Figure 1C). The zeta potentials of T‐Exo and T‐A‐Exo suspensions were −7.68 and −7.98 mV, respectively, whereas the zeta potential of T‐AC‐Exo suspension was −9.25 mV, which was higher than the former two (Figure S3B, Supporting Information). The difference in zeta potentials of the exosome suspensions might be attributed to the different numbers of exosome particles in the suspensions, i.e., the higher number of exosomes in T‐AC‐Exo suspension resulted in a larger value of the suspension zeta potential. Western blotting analysis exhibited the exosomes expressed abundant exosomal markers of tumor susceptibility gene 101 protein (TSG101), tetraspanin‐30 (CD63), and tetraspanin‐28 (CD81) with low levels of cell‐specific marker glyceraldehyde‐3‐phosphate dehydrogenase (GAPDH) (Figure 1D). The protein concentrations of the isolated exosomes derived from MΦs cultured on the specimens were in the following order: T‐AC‐Exo > T‐A‐Exo > T‐Exo (Figure 1E). Approximately 8.1 µg of exosomes (protein content) could be secreted by 1 × 10^6^ MΦs in T‐AC‐Exo group, and the isolated exosomes of T‐A‐Exo and T‐Exo groups were, respectively, 6.54 and 5.37 µg at protein levels.

### 2.2 The MΦ‐Derived Exosomes Regulated In Vivo Vascular Reconstruction and Bone Regeneration

**Figure 2:**
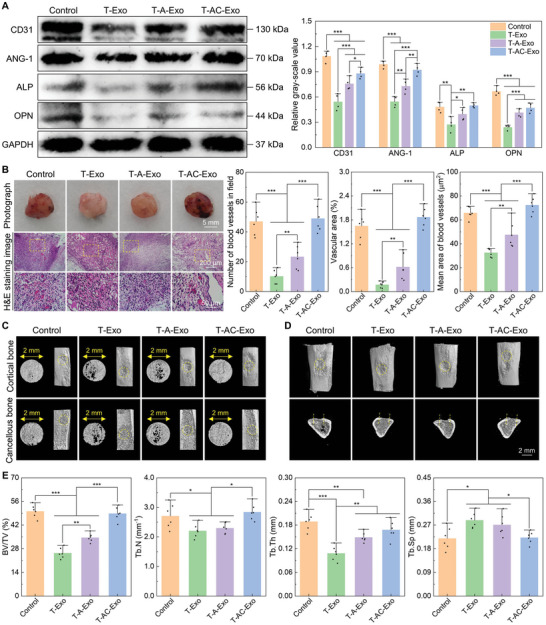
Exosomes derived from MΦs cultured on different specimen surfaces show distinct in vivo angiogenic and osteogenic activities. A) Western blotting analysis for the angiogenesis/osteogenesis‐related protein levels in newborn bone tissues (protein blotting images and quantitative analysis) (*n* = 4). B) Representative photographs and H&E staining images of Matrigel plugs and quantitative statistics of vascular structures (*n* = 6). C) Plan views for the micro‐CT reconstruction of bone defects. D) The micro‐CT reconstruction images of the tibias and tibial cross‐sections at the bone defects. E) Quantitative analysis of bone structure parameters (BV/TV, Tb.N, Tb.Th, and Tb.Sp) (*n* = 6). Data are presented as means ± SD. ^*^ *p* < 0.05, ^**^ *p* < 0.01, and ^***^ *p* < 0.001.

The tendencies of in vivo angiogenesis and osteogenesis mediated by the MΦ‐derived exosomes, as assessed by western blotting analysis, are shown in **Figure** **2**A. Compared with Control, the MΦ‐derived exosomes inhibited the protein expression of angiogenic/osteogenic‐related factors. The inhibition on protein levels of platelet and endothelial cell adhesion molecule 1 (CD31, also known as PECAM‐1), angiopoietin‐1 (ANG‐1), alkaline phosphatase (ALP), and osteopontin (OPN) was prominently reduced in T‐A‐Exo group compared with T‐Exo group. Noteworthily, the protein levels of angiogenic‐related factors CD31 and ANG‐1 in T‐AC‐Exo group were significantly enhanced than T‐Exo and T‐A‐Exo groups, while the protein levels of osteogenic‐related factors ALP and OPN were slightly higher in T‐AC‐Exo group than T‐A‐Exo group. In addition, T‐AC‐Exo mediated the protein expression of ANG‐1 and ALP at equal levels to the control group. Visual photographs of the Matrigel plugs showed that obvious red aggregated clots and capillary networks could be observed in Control and T‐AC‐Exo groups, whereas in groups T‐Exo and T‐A‐Exo fewer clots and blood vessels were observed, especially in T‐Exo group where the Matrigel plug appeared a conspicuous white color (Figure 2B). Hematoxylin and eosin (H&E) staining of the Matrigel plug sections also revealed that abundant blood vessels formed in the Control and T‐AC‐Exo groups. Quantitative statistics of vascular number and area showed that T‐Exo and T‐A‐Exo mediated significantly lower vascular number and area, whereas T‐AC‐Exo mediated equal levels of these as the control group. In addition, the number and area of blood vessels generated in Matrigel plugs were higher in T‐A‐Exo group than T‐Exo group. The Microcomputed tomography (micro‐CT) 3D reconstruction images of tibial cortical defects at 15 days postoperatively demonstrated that new bone tissues were generated from the edges of the defects toward the centers in all groups (Figure 2C,D). The new bone tissues almost filled the bone defect areas in the Control and T‐AC‐Exo groups, while partially visible defect gaps could still be observed in groups T‐Exo and T‐A‐Exo. Cross‐sectional 3D views of the bone defects also exhibited the more abundant new bone mass within the defect areas in groups Control and T‐AC‐Exo as well as the suboptimal bone mass in groups T‐Exo and T‐A‐Exo. Moreover, quantitative analysis of the new bone volume fractions and the 3D characteristic parameters of trabecular bone in bone defect areas showed that T‐Exo and T‐A‐Exo significantly inhibited in‐vivo regeneration and reconstruction of bone tissues and mediated sparser bone structures (Figure 2E). The control group exhibited a higher bone volume/tissue volume (BV/TV) fraction of 50.22% ± 3.78%, which distinctly and gradually increased in other three groups stimulated by the MΦ‐derived exosomes (25.47% ± 3.18% for T‐Exo group, 34.60% ± 2.56% for T‐A‐Exo group, and 48.83% ± 4.02% for T‐AC‐Exo group). Trabecular bone exhibited lower numbers, thinner thicknesses, and higher bone spaces in groups T‐Exo and T‐A‐Exo, with T‐Exo in particular modulating the least desirable bone structure parameters (trabecular number (Tb.N) of 2.21 ± 0.23 mm^−1^, trabecular thickness (Tb.Th) of 0.11 ± 0.02 mm, and trabecular separation (Tb.Sp) of 0.29 ± 0.03 mm). Additionally, T‐AC‐Exo mediated new bone formation and reconstruction of bone structures at the equivalent level to the control group, indicating no inhibitory effect on bone regeneration and reconstruction in vivo.

### 2.3 The MΦ‐Derived Exosomes Regulated In Vitro Angiogenesis of ECs

**Figure 3:**
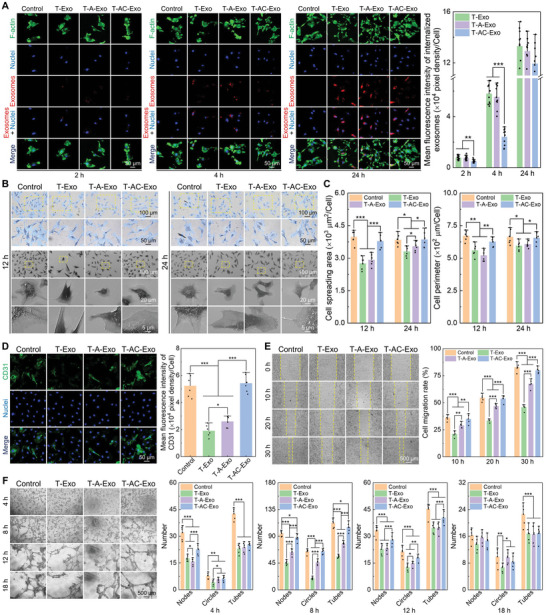
Exosomes derived from MΦs cultured on different specimen surfaces show distinct in vitro angiogenic activities. A) Exosome internalization detection and cytoskeleton assembly (fluorescence images and quantitative analysis of the exosomes taken up by cells) (*n* = 10). B) Cell morphology captured by optical microscope and FE‐SEM. C) Quantitative analysis of cellular spreading area and perimeter in FE‐SEM images (*n* = 6). D) Immunofluorescence staining images for CD31 and quantitative analysis (*n* = 6). E) Optical images of cell migration and quantitative statistics of cell migration rate (*n* = 5). F) Optical images of in vitro blood‐vessel formation and quantitative statistics of vascular network structures (*n* = 6). Data are presented as means ± SD. ^*^ *p* < 0.05, ^**^ *p* < 0.01, and ^***^ *p* < 0.001.

To investigate the biological effects of the exosomes derived from the specimen‐mediated MΦs on in vitro angiogenesis, ECs were stimulated with the exosomes. As shown in **Figure** **3**A, a handful of the PKH26‐labeled exosomes (red) were internalized into ECs after 2 h of incubation, and more exosomes were endocytosed by ECs as the increase of incubation time. After incubating for 4 h, increased and visible exosomes were localized in the partial perinuclear region. A lot of red‐fluorescent exosomes were internalized into most of the area around the nuclei after incubation for 24 h. In contrast, the negative results that no red fluorescence was shown in the control group confirmed that the appearance of red fluorescence was due to actual exosome internalization rather than excess PKH26 dye. Furthermore, there were striking differences in the number of internalized exosomes in early phases among three kinds of exosomes, suggesting that the uptake efficiencies of ECs for different exosomes were distinct. Quantitative analysis revealed that ECs took up T‐Exo and T‐A‐Exo more quickly after incubated for 2 and 4 h compared with T‐AC‐Exo, and there was no difference in cellular uptake amount of the three exosomes after 24 h of incubation. Quantitative analysis of the cytoskeleton showed that the MΦ‐derived exosomes weakened the fluorescence intensity of EC F‐actin and reduced cellular spreading area and perimeter (Figure S4A–C, Supporting Information). The negative regulations were most pronounced after 4 h of the exosome incubation and attenuated after 24 h. In addition, the MΦ‐derived exosomes suppressed initial EC adhesion, with the most prominent suppression by T‐Exo and the least by T‐AC‐Exo (Figure S4D, Supporting Information). Moreover, the MΦ‐derived exosomes did not produce cytotoxicity to ECs but inhibited EC proliferation, especially T‐Exo (Figure S4E,F, Supporting Information). The observation of EC morphology confirmed that the MΦ‐derived exosomes could stimulate cell morphology contraction and inhibit cell spreading, which echoed the results of cytoskeleton fluorescence staining (Figure 3B). The ECs stimulated by T‐Exo and T‐A‐Exo presented the contracted cell spreading area and restrained pseudopod stretching after 12 h of incubation. This phenomenon was weakened after 24 h of incubation. In addition, T‐AC‐Exo did not show an inhibitory effect on EC morphology extension. The quantitative analysis of cell spreading area and cell perimeter confirmed the above observation (Figure 3C).

The angiogenic capacities of ECs were also manipulated by the MΦ‐derived exosomes. As shown in Figure 3D, T‐Exo and T‐A‐Exo downregulated the levels of CD31 compared with Control and T‐AC‐Exo, especially T‐Exo significantly inhibited the CD31 expression of ECs. Moreover, T‐AC‐Exo did not show a downregulated effect on CD31 level. The results of the wound‐healing assay demonstrated that T‐Exo and T‐A‐Exo observably restricted the migration of ECs (Figure 3E). Notably, T‐Exo exhibited the most conspicuous inhibitory effects on EC migration, and the inhibition was attenuated by T‐A‐Exo. The migration ability of ECs showed no significant difference between the T‐AC‐Exo and Control groups. However, the MΦ‐derived exosomes remarkably inhibited in vitro blood‐vessel formation. After 8 h of incubation, the exosome stimulation resulted in the formation of sparser vascular networks compared with the well‐developed vascular network in the control group, especially T‐Exo significantly impaired angiogenic activities of ECs (Figure 3F). Quantitative results demonstrated the inhibitory effects of T‐A‐Exo on angiogenesis receded compared with T‐Exo, while T‐AC‐Exo showed the least inhibitory effects.

### 2.4 The MΦ‐Derived Exosomes Regulated In Vitro Osteogenesis of BMSCs

**Figure 4:**
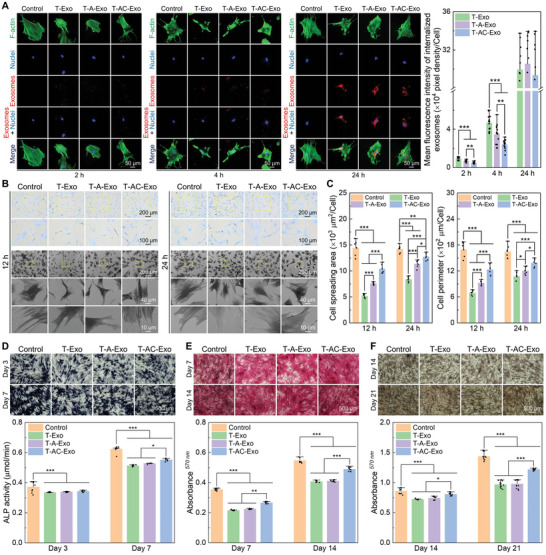
Exosomes derived from MΦs cultured on different specimen surfaces show distinct in vitro osteogenic activities. A) Exosome internalization detection and cytoskeleton assembly (fluorescence images and quantitative analysis of the exosomes taken up by cells) (*n* = 10). B) Cell morphology captured by optical microscope and FE‐SEM. C) Quantitative analysis of cellular spreading area and perimeter in FE‐SEM images (*n* = 6). D) Qualitative images and quantitative analysis for ALP activity (*n* = 9). E) Qualitative images and quantitative analysis for collagen secretion (*n* = 9). F) Qualitative images and quantitative analysis for ECM mineralization (*n* = 9). Data are presented as means ± SD. ^*^ *p* < 0.05, ^**^ *p* < 0.01, and ^***^ *p* < 0.001.

To determine the effects of the MΦ‐derived exosomes on osteogenesis, osteogenic activities of BMSCs were assessed after the exosome stimulation. Similar to ECs, small amounts of the exosomes were taken up by BMSCs at an early phase, internalized exosomes appeared in the perinuclear region after 4 h of incubation, and a large number of the exosomes were internalized into the perinuclear region after 24 h of incubation (**Figure** **4**A). Moreover, T‐Exo was internalized into BMSCs most efficiently during the early phases of the exosome incubation, followed by T‐A‐Exo. Quantitative analysis of the BMSC cytoskeleton indicated that the MΦ‐derived exosomes significantly inhibited the actin stretching and cytoskeleton extension of BMSCs, with T‐Exo exhibiting the most pronounced inhibitory effects and T‐AC‐Exo the weakest (Figure S5A–C, Supporting Information). The MΦ‐derived exosomes suppressed the initial adhesion of BMSCs, with T‐Exo showing the most significant suppressive effect and T‐AC‐Exo the least (Figure S5D, Supporting Information). The exosomes were not cytotoxic to BMSCs, but T‐Exo and T‐A‐Exo inhibited BMSC proliferation, especially T‐Exo (Figure S5E,F, Supporting Information). Observation of BMSC morphology indicated that the MΦ‐derived exosomes significantly suppressed BMSC spreading and morphology extension (Figure 4B). Compared with the favorable cell spreading state and abundant pseudopodia of BMSCs in the control group, T‐Exo significantly inhibited BMSC spreading and pseudopod stretching after 12 h of incubation, causing the cells to present a lathy morphology. Furthermore, BMSCs stimulated by T‐A‐Exo and T‐AC‐Exo for 12 h also showed restrained morphological extension, but the restraint by T‐AC‐Exo was small and the inhibitory effects by all exosomes were reduced after 24 h of incubation. Quantitative statistics of the cell morphology parameters are depicted in Figure 4C, which effectively demonstrates the inhibitory regulation of BMSC morphology sizes by the exosomes. The observation was consistent with the trend observed in the analysis of the cytoskeleton.

As a critical enzyme during osteogenesis, ALP is an early marker for osteogenic differentiation. The qualitative and quantitative results of ALP activity are shown in Figure 4D. The staining images of insoluble bluish‐violet NBT‐formazan revealed that the ALP produced by BMSCs increased with prolonged osteogenic induction, and the MΦ‐derived exosomes inhibited ALP activity as evidenced by fewer blue–purple NBT‐formazan. Quantitative results of ALP activity were mirrored with qualitative results. The ALP activity of BMSCs without the exosome stimulation was the highest at all detection time points, while the ALP activity mediated by T‐AC‐Exo was higher than that of the other two exosome groups after 7 days of osteogenic induction. Collagen is the main component of bone extracellular matrix (ECM), and its secretion is regarded as a marker for the middle stage of osteogenic differentiation. The collagen dyed deep red became more in amount as increased time of osteogenic induction (Figure 4E). The secreted collagen was scarcer from BMSCs stimulated by the MΦ‐derived exosomes than that without exosome stimulation at each time point, while T‐AC‐Exo induced more collagen secretion compared with T‐Exo and T‐A‐Exo. Meanwhile, the results of ECM mineralization based on Alizarin Red S staining showed a similar trend (Figure 4F). Optical images exhibited more visible brown mineralized nodules after 21 days of osteogenic induction compared with 14 days. The exosomes derived from MΦs exhibited a prominent inhibition of mineralized nodule formation compared with Control, whereas T‐AC‐Exo exerted a relatively weaker inhibitory effect on ECM mineralization in comparison with T‐Exo and T‐A‐Exo.

### 2.5 The MΦ‐Derived Exosomes Induced the Internalization, Trafficking, and Degradation of Integrin β1 in Recipient Cells

**Figure 5:**
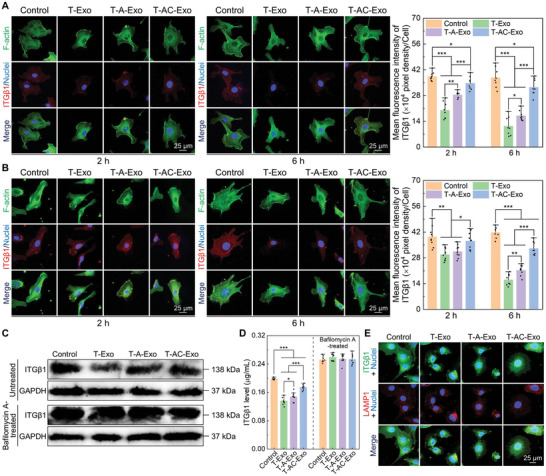
Exosomes derived from MΦs cultured on different specimen surfaces differently regulate internalization and targeted lysosomal degradation of membrane ITGβ1 of ECs. A) Immunofluorescence staining images of membrane ITGβ1 (omitted cell permeabilization) and quantitative analysis (*n* = 8). B) Immunofluorescence staining images of total ITGβ1 and quantitative analysis (*n* = 8). C) Western blotting analysis for cellular total ITGβ1 levels after incubated with the exosomes for 6 h (bafilomycin A was used to inhibit lysosomal degradation). D) ELISA test for cellular total ITGβ1 levels after incubated with the exosomes for 6 h (*n* = 9). E) Colocalization analysis of ITGβ1 with LAMP1 after incubated with the exosomes for 4 h. Data are presented as means ± SD. ^*^ *p* < 0.05, ^**^ *p* < 0.01, and ^***^ *p* < 0.001.

Immunofluorescence staining assay revealed that the MΦ‐derived exosomes mediated the internalization of membrane integrin β1 (ITGβ1) into ECs and effectively decreased the levels of the membrane ITGβ1 and total ITGβ1 (**Figure** **5**A,B). After 2 h of the exosome incubation, the membrane ITGβ1 of ECs stimulated by the exosomes was decreased and the partially internalized ITGβ1 had been transported to the perinuclear region. After 6 h of incubation, the exosome‐mediated internalization of membrane ITGβ1 was more pronounced, while the internalized ITGβ1 mediated by T‐Exo and T‐A‐Exo was remarkably clustered in the perinuclear region. Quantitative analysis of ITGβ1 levels substantiated that the MΦ‐derived exosomes mediated internalization and reduction of ITGβ1, while T‐AC‐Exo exhibited a lesser effect. To investigate further regulation of the internalized ITGβ1 by the MΦ‐derived exosomes, bafilomycin A was used to inhibit cellular lysosomal degradation. As shown in Figure 5C,D, the exosomes significantly decreased total ITGβ1 levels of ECs after 6 h of incubation, and the reduction was blocked by pretreatment with bafilomycin A. Moreover, enzyme‐linked immunosorbent assay (ELISA) results manifested a quantitative distinction in the ITGβ1 levels regulated by three groups of exosomes, with T‐Exo mediating the most conspicuous reduction of ITGβ1, whereas T‐AC‐Exo mediating the least reduction. Additionally, the exosome stimulation caused restricted cell spreading and cell morphology contraction regardless of pretreatment with bafilomycin A (Figure S6, Supporting Information). The above suggested that the MΦ‐derived exosome‐induced reduction in cellular ITGβ1 was associated with the lysosomal degradation process. Immunofluorescent labeling showed that the exosome stimulation increased the colocalization of ITGβ1 with lysosomal‐associated membrane protein 1 (LAMP1)‐positive lysosomes in ECs, while the internalized ITGβ1 and lysosomes clustered in the perinuclear region (Figure 5E). This indicated that the MΦ‐derived exosomes induced ITGβ1 trafficking to lysosomes and resulted in the degradation of the internalized ITGβ1. Consistent with ECs, the results presented in Figures S7 and S8 (Supporting Information) revealed that the MΦ‐derived exosomes similarly induced the internalization and targeted lysosomal degradation of membrane ITGβ1 in BMSCs. T‐Exo‐mediated ITGβ1 internalization and degradation were the most prominent, while T‐AC‐Exo‐mediated effects were less pronounced.

### 2.6 Deficiency of Integrin β1 Impaired the Angiogenic Capacity of ECs

**Figure 6:**
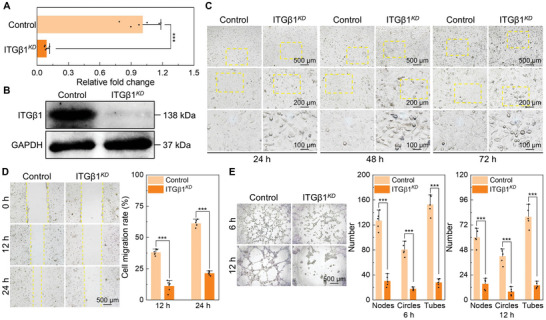
Knockdown of ITGβ1 impairs angiogenic capacity of ECs. A) Gene expression of ITGβ1 in normal (Control) and siRNA‐mediated ITGβ1‐knockdown (ITGβ1*^KD^*) ECs detected by qRT‐PCR after transient transfection for 24 h (*n* = 6). B) Protein expression of ITGβ1 analyzed by western blotting after transient transfection for 72 h. C) Cell morphology of normal and ITGβ1*^KD^* ECs after transient transfection for 24, 48, and 72 h. D) Migration behaviors of normal and ITGβ1*^KD^* ECs (optical images and quantitative statistics of cell migration rate) (*n* = 6). E) In vitro blood‐vessel formation of normal and ITGβ1*^KD^* ECs (optical images of vascular network and quantitative statistics of vascular network structures) (*n* = 5). Data are presented as means ± SD. ^***^ *p* < 0.001.

To investigate the association between the ITGβ1 and angiogenic capacity of ECs, a transient transfection approach utilizing small interfering RNA (siRNA) was implemented to silence ITGβ1 gene expression in ECs. The successful knockdown of ITGβ1 (ITGβ1*^KD^*) was verified through qRT‐PCR and western blotting, showing the remarkable reduction in both mRNA and protein levels of ITGβ1 in ECs after siRNA transfection (**Figure** **6**A,B). As shown in Figure 6C, the ITGβ1*^KD^* ECs exhibited curled morphology and restricted cell spreading compared with the control group. Knockdown of ITGβ1 severely impaired EC migration capacity (Figure 6D). Furthermore, in vitro blood‐vessel formation of ITGβ1*^KD^* ECs was markedly reduced than that of the normal cells (Figure 6E). All of the above highlighted the crucial roles of ITGβ1 in regulating cell spreading and function of ECs, with its deficiency significantly compromising cellular angiogenic capacity.

### 2.7 Extracellular Microenvironmental Cytokines Binding to the Outer Membrane of the MΦ‐Derived Exosomes Determined Their Regulatory Functions

**Figure 7:**
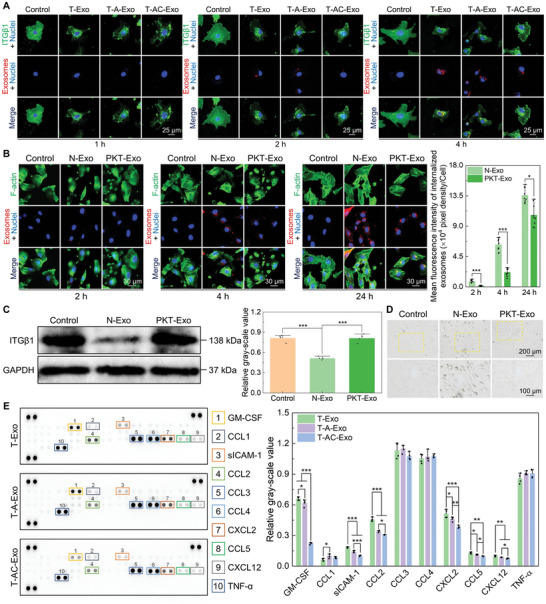
Screening of key cytokines on the exosomes involved in the immunomodulation on angiogenesis and osteogenesis. A) Colocalization analysis of ITGβ1 with internalized exosomes in ECs. B) Exosome internalization detection of normal exosomes (N‐Exo) and Proteinase K‐treated exosomes (PKT‐Exo) in ECs (fluorescence images and quantitative analysis of the exosomes taken up by cells) (*n* = 9). C) Western blotting analysis for total ITGβ1 levels in ECs mediated by N‐Exo and PKT‐Exo after 6 h of incubation (protein blotting images and quantitative analysis) (*n* = 4). D) Cell morphology of ECs mediated by N‐Exo and PKT‐Exo after 6 h of incubation. E) Cytokine array analysis of the exosome suspensions (spot blotting images of cytokines and quantitative analysis) (*n* = 4). Data are presented as means ± SD. ^*^ *p* < 0.05, ^**^ *p* < 0.01, and ^***^ *p* < 0.001.

To detect whether ITGβ1 was internalized along with the exosomes, colocalization analysis of the internalized ITGβ1 and exosomes through immunofluorescence staining assays was performed. As shown in **Figure** **7**A, the internalized ITGβ1 were not colocalized with the MΦ‐derived exosomes in ECs and the internalization process of ITGβ1 preceded that of the exosomes. After 1 h of exosome stimulation, partially internalized ITGβ1 had been transported to the perinuclear region, but no visible exosome taken up by ECs was observed. After 2 h of stimulation, a large amount of internalized ITGβ1 was aggregated in the perinuclear region, while a small amount of the exosomes was localized at the plasma membrane region of ECs. After 4 h of stimulation, the fluorescence intensity of the internalized ITGβ1 mediated by the exosomes was significantly reduced, indicating that the ITGβ1 had begun to degrade, especially that mediated by T‐Exo and T‐A‐Exo, while the exosomes had only just been internalized into the perinuclear region. Colocalization analysis similarly revealed that the ITGβ1 internalization was faster than the exosome internalization in BMSCs (Figure S9A, Supporting Information).

To ascertain whether regulatory factors of the MΦ‐derived exosomes were localized within the exosome or on the exosome outer membrane, Proteinase K was used to treat the exosome to degrade extramembrane proteins. T‐Exo was chosen for subsequent experiments as representative MΦ‐derived exosomes (normal exosomes, denoted as N‐Exo or Exo), while Proteinase K‐treated T‐Exo were denoted as PKT‐Exo. Exosome internalization assays manifested that PKT‐Exo was still taken up by ECs, but the internalization of PKT‐Exo was much slower than that of N‐Exo (Figure 7B). After 2 h of the exosome incubation, a small amount of internalized N‐Exo could be seen, but no PKT‐Exo was detected. After 4 h of incubation, a handful of internalized PKT‐Exo were located in the cytoplasmic region, while more N‐Exo had been internalized into the perinuclear region. A large number of internalized N‐Exo and PKT‐Exo converged at the perinuclear region after 24 h of incubation, but the number of internalized N‐Exo was still greater than PKT‐Exo. Western blotting analysis indicated that PKT‐Exo did not mediate a reduction in ITGβ1 levels of ECs, whereas N‐Exo induced obvious ITGβ1 degradation (Figure 7C). The morphology and spreading state of ECs stimulated by PKT‐Exo were comparable to those in the control group, which significantly differed from the contracted cell morphology and restrained cell spreading induced by N‐Exo (Figure 7D). Similar to ECs, PKT‐Exo was taken up by BMSCs at a lower rate than N‐Exo and it did not cause ITGβ1 degradation and cell morphology contraction (Figure S9B–D, Supporting Information). Therefore, the proteins located on the exosome outer membrane might induce the internalization and degradation of ITGβ1 in recipient cells.

**Figure 8:**
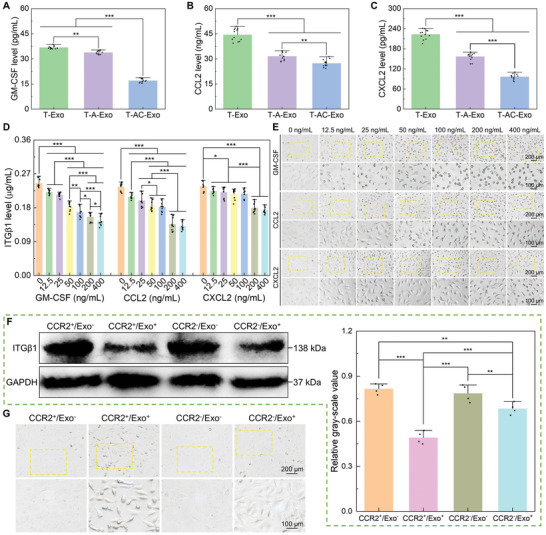
Biomaterial surfaces can vary the content of exosomal CCL2 of MΦs to regulate membrane ITGβ1 trafficking and therefore the functions of recipient cells. A–C) Quantitative analysis for levels of the exosome‐bound GM‐CSF, CCL2, and CXCL2 determined by ELISA (*n* = 12). D) ELISA test for cellular total ITGβ1 levels of ECs after stimulated with gradient concentrations of GM‐CSF, CCL2, and CXCL2 for 6 h (*n* = 9). E) Cell morphology of ECs after stimulated with GM‐CSF, CCL2, and CXCL2 for 6 h. F) Western blotting analysis for total ITGβ1 levels of ECs mediated by exosome/CCR2 binding after incubated with the exosomes for 6 h (protein blotting images and quantitative analysis) (*n* = 4). G) Cell morphology mediated by exosome/CCR2 binding after incubated with the exosomes for 6 h. Data are presented as means ± SD. ^*^ *p* < 0.05, ^**^ *p* < 0.01, and ^***^ *p* < 0.001.

Cytokine arrays incubated with the exosome suspensions revealed that the isolated exosomes carried a diverse range of cytokines (Figure 7E). Specifically, C‐C motif chemokine ligand 3 (CCL3), CCL4, and TNF‐α exhibited the highest abundances on the exosomes, while granulocyte‐MΦ colony‐stimulating factor (GM‐CSF), CCL2, and C‐X‐C motif chemokine ligand 2 (CXCL2) possessed relatively high abundances and exhibited significant differences in enrichment on the three groups of exosomes. Therefore, GM‐CSF, CCL2, and CXCL2 might be key regulators of the three MΦ‐derived exosomes causing distinct regulations. ELISA tests showed a sequential decrease in the bound amounts of GM‐CSF, CCL2, and CXCL2 on T‐Exo, T‐A‐Exo, and T‐AC‐Exo (**Figure** **8**A–C). On 10 µg mL^−1^ of exosomes, the amounts of GM‐CSF bound to the three groups of exosomes were ≈36.78, 33.81, and 17.00 pg mL^−1^, the amounts of bound CCL2 were ≈44.38, 31.51, and 27.35 ng mL^−1^, and the amounts of bound CXCL2 were ≈223.12, 156.98, and 96.48 pg mL^−1^, respectively. Proteinase K treatment led to dramatic degradation of the cytokines bound to the exosome outer membrane, while the concentrations of PKT‐Exo‐bound GM‐CSF, CCL2, and CXCL2 were almost zero (Figure S9E–G, Supporting Information).

The gene expression of ten cytokines screened by the cytokine array assay exhibited high levels in the specimen‐mediated MΦs (Figure S10A, Supporting Information). Particularly, the gene expression levels of GM‐CSF, CCL2, and CXCL2 differed significantly in the MΦs mediated by the specimens T, T‐A, and T‐AC and tended to decrease sequentially. Moreover, the concentrations of the three cytokines secreted by the specimen‐mediated MΦs into supernatants presented higher levels and decreased sequentially among the groups T, T‐A, and T‐AC (Figure S10D–F, Supporting Information). Therefore, concentration differences of GM‐CSF, CCL2, and CXCL2 in the extracellular microenvironment of the specimen‐mediated MΦs might lead to differences in the exosome‐bound amounts, thereby resulting in different manipulation of ITGβ1 and cellular functions by T‐Exo, T‐A‐Exo, and T‐AC‐Exo. Nanostructure and Col‐I modulated inflammatory responses and polarization of MΦs by controlling signal transduction, and the specimens T, T‐A, and T‐AC exhibited a gradient‐trending regulation on the expression of key signaling cascade genes (Figure S10B,C, Supporting Information). The MΦs mediated by T‐AC exhibited the highest expression levels of integrin‐related genes, focal adhesion kinase (FAK), phosphoinositide‐3‐kinase (PI3K), protein kinase B (AKT), mammalian target of rapamycin (mTOR), and cAMP‐response element binding protein (CREB), which are involved in cell adhesion and PI3K‐AKT signaling pathway. In addition, T‐AC‐mediated MΦs expressed the highest levels of growth factors VEGF, BMP2, leukemia inhibitory factor (Lif), oncostatin M (Osm), and PDGF, which are transcriptionally regulated by the PI3K‐AKT signaling pathway. Moreover, T‐AC significantly downregulated the expression of Toll‐like receptor (TLR‐4), CD14, Tat‐associated kinase (TAK), TGF‐β‐activated kinase (TAB), nuclear factor‐κB (NF‐κB), and inhibitor of NF‐κB (I‐κB), which participate in Toll‐like receptor and NF‐κB signaling pathways.

### 2.8 Exosome‐Bound CCL2 Acted as a Major Regulator Mediating the Integrin β1 Internalization and Degradation

The exosome (T‐Exo)‐bound GM‐CSF detected by ELISA was ≈36.78 pg mL^−1^, CCL2 was ≈44.38 ng mL^−1^, and CXCL2 was ≈223.12 pg mL^−1^ (Figure 8A–C). The GM‐CSF, CCL2, and CXCL2 with increasing concentration gradients were used to directly stimulate cells to investigate their effects on cellular ITGβ1 levels. As shown in Figure 8D; and Figure S12A (Supporting Information), ITGβ1 levels of ECs and BMSCs exhibited a concentration‐dependent decrease of GM‐CSF, CCL2, and CXCL2. As the action concentration of the cytokines increased, the degradation of ITGβ1 intensified, thus causing cell spreading restricted and morphology contracted of recipient cells (Figure 8E; and Figure S12B (Supporting Information)). Based on comparisons with the cytokine concentration gradients, the exosome‐bound CCL2 was sufficient to induce significant degradation of ITGβ1 in ECs and BMSCs, whereas the levels of the exosome‐bound GM‐CSF and CXCL2 were insufficient to cause a decrease in ITGβ1 levels. Therefore, it could be inferred that the exosome‐bound CCL2 played the most crucial role in the internalization, trafficking, and degradation of ITGβ1 regulated by the MΦ‐derived exosomes. C‐C motif chemokine receptor 2 (CCR2) is the main functional receptor for CCL2. Upon antagonizing the binding activity of CCR2 in recipient cells, CCL2 no longer mediated a reduction in ITGβ1 levels nor did it cause cell spreading restriction and morphology contraction (Figure S11; and Figure S12C,D, Supporting Information). Additionally, the exosome‐mediated ITGβ1 degradation, cell spreading restriction, and cell morphology contraction were significantly alleviated upon inhibition of CCR2 binding activity (Figure 8F,G; and Figure S12E,F (Supporting Information)). However, relatively obvious ITGβ1 level reduction and cell spreading restriction were still visible in group CCR2^−^/Exo^+^ compared with the group not stimulated by the exosomes (CCR2^+^/Exo^−^ and CCR2^−^/Exo^−^). The MΦ‐derived exosomes significantly upregulated cellular autophagosome levels and there was no obvious difference in the exosome‐mediated autophagy levels among T‐Exo, T‐A‐Exo, and T‐AC‐Exo, while CCL2‐mediated autophagosome levels were the same as the control group (Figure S13, Supporting Information).

## 3 Discussion

Cytokines secreted by MΦs mediated by the physicochemical properties of the implant surfaces are thought to exert primary mechanisms of immunomodulation.^[^ ^12^, ^13^ ^]^ However, as key mediators of intercellular communication, the roles of exosomes in immunomodulation have become prominent. In this study, we reported that Ti implant‐mediated MΦ‐derived exosomes significantly impeded vascularization and bone regeneration in vivo and inhibited the functions of ECs and BMSCs in vitro. These observations could be ascribed to MΦ‐derived exosome‐regulated internalization and targeted lysosomal degradation of membrane ITGβ1 of recipient cells. Protease K treatment assay and proteomic analysis revealed that inflammatory cytokines bound to the MΦ‐derived exosome outer membrane manipulated the internalization and degradation processes of ITGβ1 in recipient cells, with CCL2 playing the most important regulatory roles. Col‐I‐decorated nanostructured surfaces mediated low levels of inflammatory cytokines bound to the MΦ‐derived exosomes (T‐AC‐Exo), which consequently regulated ITGβ1 degradation to a low degree and inhibited cellular functions to a less extent when compared with that of the untreated surface group.

Integrins, the heterodimers composed of α‐ and β‐subunits, are major cell surface receptor proteins that play a fundamental role in interacting with ECM and transmitting extracellular signals.^[^ ^25^, ^26^ ^]^ They can be internalized by clathrin‐dependent and ‐independent pathways and delivered into Rab5‐positive early endosomes (EEs), after which it is sorted and recycled back to the plasma membrane through two distinct pathways (short loop and long loop).^[^ ^26^ ^]^ In the case of ITGβ1, protein kinase D1 (PKD1) binds to the C‐terminal region of ITGβ1 cytodomain and traffics EEs to the cell membrane to achieve the ITGβ1 recycling during short loop recycling.^[^ ^27^ ^]^ During long loop recycling, sorting nexin 17 (SNX17) localizes to EEs, binds its FERM structural domain to the NPxY motif at ITGβ1 cytodomain tails, and subsequently traffics the endocytosed ITGβ1 to Rab11‐positive recycling endosomes (REs) and recycles it to the plasma membrane.^[^ ^28^, ^29^ ^]^ The internalized ITGβ1 can also be degraded through two pathways (autophagic degradation and endocytic degradation). During autophagic degradation, autophagy proteins UNC‐51‐like kinase 1 (ULK1) and autophagy‐related protein 9 (ATG9) localize to the REs, deliver the endocytosed integrins to expanding phagophores, and initiate autophagosome assembly.^[^ ^30^, ^31^ ^]^ Subsequently, autophagosomes fuse with lysosomes to form autolysosomes, in which the cargoes are degraded. During endocytic degradation, extracellular signals stimulate integrin ubiquitination, and the ubiquitinated integrins are endocytosed into EEs and then sorted into multivesicular endosomes (MVEs), followed by trafficked into Rab7‐positive late endosomes (LEs) which fuse with lysosomes for integrin degradation.^[^ ^26^, ^32^, ^33^ ^]^ The recycling process rearranges the distribution of membrane integrins and manipulates the cell spreading and migration behavior. ITGβ1 is the most widely expressed integrin subunit and the majority of integrins contain the β1‐subunit.^[^ ^26^ ^]^ Regular turnover of ITGβ1 via the endocytic‐exocytic pathway determines cell adhesion, spreading, migration, and motility.^[^ ^26^, ^34^ ^]^ Specifically, ITGβ1 is the crucial regulator for the angiogenesis of ECs and osteogenesis of BMSCs.^[^ ^35^, ^36^, ^37^, ^38^ ^]^ Our data also showed that the loss of ITGβ1 resulted in a serious decline in the migratory capacity and angiogenic activity of ECs (Figure 6). The MΦ‐derived exosomes regulated ITGβ1 internalization in recipient cells, and the internalized ITGβ1 was not recycled back to the plasma membrane but accumulated in the perinuclear region and transported to the lysosome for degradation (Figure 5; and Figure S7 (Supporting Information), and Figure S8 (Supporting Information)). These resulted in ITGβ1 deficiency on the recipient cell membranes, which caused restricted cell spreading and curled‐up cell morphology, thereby inhibiting cell proliferation and differentiation (Figures 3 and 4; and Figures S4 and S5 (Supporting Information)). Animal experiments demonstrated that the MΦ‐derived exosomes similarly inhibited angiogenesis and osteogenesis, especially T‐Exo, which was also supposed to be related to the exosome‐mediated degradation of ITGβ1 in tissue cells (Figure 2). The PKT‐Exo was still able to be taken up by recipient cells, but was internalized less efficiently than the N‐Exo and did not mediate a decrease in ITGβ1 levels (Figure 7B,C; and Figure S9B,C (Supporting Information)), suggesting the critical components of the MΦ‐derived exosomes that regulate ITGβ1 internalization and degradation were located at the outer membrane. Recipient cells take up exosomes by numerous routes, among which internalization of exosomes via cell surface binding to exosome surface proteins (including tetraspanins, integrins, extracellular matrix proteins, and proteoglycans) is an essential manner.^[^ ^15^, ^17^ ^]^ Proteinase K degraded the exosome surface proteins, thereby affecting exosome internalization by surface‐binding, which resulted in reduced efficiency of early PKT‐Exo internalization. However, the PTK‐Exo could also be taken up by cells through other pathways such as membrane fusion, phagocytosis, micropinocytosis, and endocytosis. ITGβ1 internalization was faster and greatly preceded exosome internalization (Figure 7A; and Figure S9A (Supporting Information)), supporting the binding of the exosome surface proteins to the membrane receptors in a ligand/receptor‐dependent manner, which triggered downstream signaling pathways that regulated ITGβ1 internalization and degradation.

**Figure 9:**
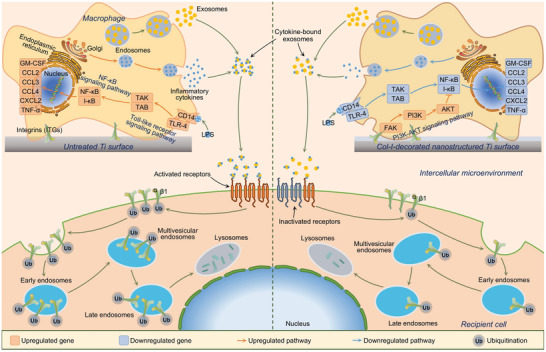
Schematic illustration of the potential mechanisms by which the biomaterial surfaces regulated the compositions of MΦ‐derived exosomes and the exosomes induced differential trafficking of membrane ITGβ1 in recipient cells.

**Figure** **9** depicts the potential mechanisms of the implant surface varied the composition of MΦ‐derived exosomes, which in turn mediated ITGβ1 internalization and degradation of recipient cells. Nanostructure and Col‐I facilitated cell adhesion and podosome formation of MΦs, thereby initiating FAK‐mediated PI3K‐AKT signaling pathway that promoted the expression of multiple growth factors and suppressed NF‐κB signal transduction (Figures S2 and S10, Supporting Information). The growth factors (such as VEGF, TGF‐β, and PDGF) secreted by MΦs further enhanced the PI3K‐AKT signaling cascade through autocrine feedback loops. The upregulated PI3K‐AKT signaling pathway and downregulated NF‐κB signaling pathway drove MΦ polarization from the M1 phenotype toward the M2 phenotype, significantly reducing the production and release of proinflammatory cytokines. In addition to soluble cytokines, the exosomes were also secreted by MΦs into the extracellular microenvironment. In addition to the different biogenesis and release amounts of exosomes mediated by the specimens, the three sets of specimens mediated MΦs to secrete different amounts of exosomes based on protein levels possibly due to the significant differences in the protein composition of the exosomes (Figure 1E). Cytokine arrays revealed that the MΦ‐derived exosomes carried an abundance of inflammatory cytokines such as GM‐CSF, CCL2, CCL3, CCL4, CXCL2, and TNF‐α (Figure 7E). It has been reported that microenvironmental cytokines bind to the external surface of exosomes via glycosaminoglycan side chains of proteoglycans on the exosome membranes and the binding increases in a cytokine concentration‐dependent manner.^[^ ^39^ ^]^ The most remarkable differences in the levels of GM‐CSF, CCL2, and CXCL2 bound to the three groups of the exosomes were observed, which might be due to the significant differences in the levels of GM‐CSF, CCL2, and CXCL2 secreted into the extracellular microenvironment by the three specimen surface‐mediated MΦs (Figure 7E, Figure 8A; and Figure S10 (Supporting Information)). Among them, the concentration of the exosome‐bound CCL2 was the highest and within the effective range of action. As a typical G‐protein‐coupled receptor, CCR2 is the main functional receptor for CCL2.^[^ ^40^ ^]^ Antagonizing the binding activity of CCR2 in recipient cells dramatically mitigated the exosome‐mediated reduction in ITGβ1 levels (Figure 8F; and Figure S12E (Supporting Information)). However, CCR2 antagonism did not completely eliminate the exosome‐induced ITGβ1 degradation, which might be caused by other exosome‐bound inflammatory cytokines. Taken together, the above results suggested that CCL2 was the predominant factor regulating the internalization, trafficking, and degradation of ITGβ1. Furthermore, previous study has shown that the Col‐I‐decorated nanostructured surface‐mediated MΦ immune microenvironment significantly promoted angiogenesis and osteogenesis.^[^ ^4^ ^]^ However, Col‐I‐decorated nanostructured surface‐mediated MΦ‐derived exosomes exhibited a certain inhibitory effects on angiogenesis and osteogenesis, suggesting that the exosome outer membrane bound more inflammatory cytokines than growth factors, which played major regulatory roles. Moreover, the immunomodulatory effects of soluble cytokines in the implant‐mediated MΦ immune microenvironment may be greater than that of the MΦ‐derived exosomes.

CCL2/CCR2 axis activates phospholipase C (PLC) and triggers diacylglycerol (DAG) messenger signaling, which targets protein kinase Cα (PKCα) and mediates its activation.^[^ ^34^, ^41^, ^42^ ^]^ Activated PKCα promotes formin‐like 2 (FMNL2) phosphorylation and manipulates its interaction with the integrin subunit tail to drive ITGβ1 internalization. We found that the internalized ITGβ1 was trafficked to the perinuclear region and degraded by lysosomes, rather than being transported to REs and recycled back to the plasma membrane. As mentioned earlier, ITGβ1 can be degraded through autophagic and endocytic pathways. However, it was found that the MΦ‐derived exosomes enhanced autophagy in recipient cells and there was no difference in the level of cellular autophagy mediated by three groups of the exosomes, whereas CCL2 exhibited no effect on autophagy (Figure S13, Supporting Information), indicating that the CCL2‐mediated ITGβ1 degradation might rely on the endocytic pathway rather than an autophagic pathway. Collectively, MΦ‐derived exosomes induced recipient cell membrane ITGβ1 internalization and lysosome‐dependent degradation via their outer membrane‐bound inflammatory cytokines, with CCL2 playing a key role. The CCL2‐activated PLC‐DAG‐PKCα‐FMNL2 signaling axis regulated ITGβ1 internalization and mediated trafficking of the internalized ITGβ1 to lysosomes for degradation via the endocytic pathway (EEs‐MVEs‐LEs) (Figure 9).

Typical M1/M2 MΦ‐derived exosomes exert profound effects on angiogenesis and osteogenesis through delivering the contents.^[^ ^18^, ^19^, ^20^, ^21^ ^]^ In addition, the MΦ‐derived exosomes regulated by implant surface structures exhibited remarkable differential modulation of angiogenesis and osteogenesis. Exosomes secreted by MΦs cultured on larger‐sized TiO_2_ nanotubes deliver miR‐3473e to upregulate AKT expression in recipient cells, thereby promoting angiogenesis of ECs and osteogenesis of BMSCs.^[^ ^43^ ^]^ Compared with micrometer‐scale biphasic calcium phosphate (BCP) ceramics, submicron‐scale BCP ceramics‐stimulated MΦ‐derived exosomes facilitate osteogenic differentiation of BMSCs by transmitting miR‐142a‐5p.^[^ ^44^ ^]^ Previous studies have tended to focus on exosomal contents, especially nucleic acids including mRNAs, miRNAs, and noncoding RNAs. It takes a long time for them to be released into recipient cells and exert regulatory effects. This study presents a rapid regulatory mechanism of exosomes through the binding of the exosome outer membrane‐bound cytokines to recipient cell surface proteins. Furthermore, it is manifested that inflammatory cytokines in the extracellular microenvironment can concentration‐dependently decorate MΦ‐derived exosomes, thereby allowing the exosomes to trigger inflammatory stress responses in the recipient cells, such as internalization and degradation of membrane integrins. The surface physicochemical characteristics of the implant material determine the protein components on the surface of MΦ‐derived exosomes, thus influencing their functional features. These findings broaden the horizons of interactions between biomaterials and host immune system and provide insights for the design of advanced biomaterials from the perspective of immunomodulation. Although our study provides evidences that biomaterial surfaces can vary the quantities of inflammatory cytokines especially CCL2 on exosomes, which in turn mediate ITGβ1 trafficking and therefore functions of recipient cells, the exact trafficking mechanism is not well understood. Furthermore, we only show that the exosomes could mediate ITGβ1 internalization and targeted lysosomal degradation of recipient cells, whether the trafficking of other integrin β subunits is also mediated by the exosomes is not explored. High‐throughput omics analysis of the active components of exosomes and the exploration of their precise regulatory mechanisms on recipient cells are potential directions in the field of regulatory roles and mechanism studies of exosomes in biomaterial‐mediated cell microenvironments.

## 4 Conclusion

The MΦ‐derived exosomes mediated by biomaterial surface induced the internalization and lysosomal‐dependent degradation of membrane ITGβ1 in recipient cells, thus inhibiting their functions. Exosome surface‐bound inflammatory cytokines, especially CCL2, regulated the ITGβ1 internalization and targeted lysosomal degradation. Biomaterial surface characteristics could alter the content of CCL2 on the MΦ‐derived exosomes, thereby determining their immunomodulatory functions.

## 5 Experimental Section

### Ethical Statement

All animal experiment procedures and surgical protocols in this study were approved by the Biological and Medical Ethics Committee of Taiyuan University of Technology (No. TYUT2023051001), and performed following international standards on animal welfare.

### Fabrication and Characterization of the Specimens

Pure titanium (Ti) sheets (99.89% purity, 0.2 mm in thickness) were tailored into the discs with a diameter of 34 mm and the Ti discs were denoted as T. Subsequently, the T specimens were immersed in 5 m NaOH at 80 °C for 12 h and the alkali‐treated Ti substrates were denoted as T‐A. Finally, the T‐A specimens were immersed in 1 mg mL^−1^ type I collagen (Col‐I)/acetic acid (5 mm) solution for 12 h, which contained 2.5 mg mL^−1^ 1‐ethyl‐3‐(3‐dimethylaminopropyl) carbodiimide and 0.63 mg mL^−1^ N‐hydroxysuccinimide. These Col‐I decorated specimens were denoted as T‐AC. The surface topographies of different specimens were observed using a field‐emission scanning electron microscope (FE‐SEM, JSM‐6700F, JEOL, Japan). To quantitatively evaluate the amount of immobilized Col‐I, the specimens were immersed in 1 mL of 1% sodium dodecyl sulfate solution for 12 h to detach adsorbed Col‐I from the specimen surfaces, and the resulting solutions were collected to quantify the Col‐I concentration using a Bicinchoninic Acid (BCA) Protein Assay Kit (Sangon Biotech, China). Surface chemical compositions of the specimens were analyzed by diffuse reflectance Fourier transform infrared spectroscope (ALPHA II, Bruker Optics, Germany) and X‐ray photoelectron spectroscope (Thermo Fisher Scientific, America).

### Cell Culture

Murine‐derived RAW 264.7 macrophages (MΦs), human umbilical vein cell line EA. hy926 endothelial cells (ECs), and Sprague Dawley (SD) rat‐derived bone marrow mesenchymal stem cells (BMSCs) were supplied by the Cell Bank of the Chinese Academy of Sciences. MΦs were cultured in complete high‐glucose Dulbecco's Modified Eagle Medium (DMEM, Gibco, America) containing 10% fetal bovine serum (FBS, Gibco, America) and 1% antibiotic‐antimycotic solution (AAS) under standard humidified condition (37 °C and 5% CO_2_) and seeded on the specimen surfaces at a density of 1 × 10^5^ cells cm^−2^ unless otherwise specified. For MΦ polarization, 1 µg mL^−1^ of lipopolysaccharide (LPS, Beyotime, China) was added to the culture medium to stimulate MΦs for 2 h after 24 h of normal culture, and then the medium was replaced with serum‐free medium to incubate MΦs for an additional 12 h.^[^ ^4^ ^]^ ECs were cultured in complete DMEM containing 10% exosome‐deprived FBS (VivaCell Biosciences, China) and 1% AAS. BMSCs were cultured in complete Minimum Essential Medium Alpha Medium (α‐MEM, Gibco, America) supplemented with 10% exosome‐deprived FBS and 1% AAS and applied for inoculation at the population of 3–4 passages. If not specified otherwise, ECs and BMSCs were seeded in 24‐well cell culture plates at a density of 2 × 10^4^ cells cm^−2^ and cultured for 24 h to fully adhere and spread before exosome induction.

### Cell Viability, Proliferation, Cytoskeleton Assembly, Morphology, and Phenotype Polarization of MΦs Mediated by the Specimens

Cell viability, proliferation, cytoskeleton assembly, and morphology were detected according to the methods described in a previous study.^[^ ^4^ ^]^ Live/Dead viability/cytotoxicity kit (Invitrogen, America) and 3‐(4, 5‐dimethylthiazol‐2‐yl)‐2, 5‐diphenyltetrazolium bromide solution (MTT, Sigma‐Aldrich, America) were used to evaluate vitality and proliferation of the LPS‐activated MΦs. Fluorescent images were captured by a confocal laser scanning microscope (CLSM, C2 Plus, Nikon, Japan). Cytoskeleton assembly was counterstained by FITC‐Phalloidin and DAPI (Sigma‐Aldrich, America). The morphology of MΦs cultured on the specimens was imaged by FE‐SEM after the MΦs were fixed with 2.5% glutaraldehyde, dehydrated in the gradient ethanol (50%, 60%, 70%, 80%, 90%, 95%, and 100%), and gold‐coated. Immunofluorescence staining assay was adopted to evaluate MΦ polarization. The LPS‐activated MΦs cultured on different specimens were fixed with 4% paraformaldehyde (PFA), permeabilized, and blocked with 1% bovine serum albumin (BSA), followed by incubated with anti‐iNOS antibody (ab178945, Abcam, Britain, diluted at 1:1000) and anti‐CD206 antibody (ab64693, Abcam, Britain, diluted at 1:2000) overnight at 4 °C. Subsequently, secondary antibodies of Goat Anti‐Rabbit IgG H&L (Alexa Fluor 555) (ab150078, Abcam, Britain, diluted at 1:1000) and Goat Anti‐Rabbit IgG H&L (Alexa Fluor 488) (ab150077, Abcam, Britain, diluted at 1:1000) were applied to incubate the cells at room temperature for 1 h to combine the primary antibody, respectively. Cell nuclei were counterstained with DAPI and fluorescent images were captured using the CLSM. Semiquantitative analysis of mean fluorescence intensity (MFI) of the labeled markers for a single cell was performed by ImageJ software.

### Exosome Isolation and Characterization

Exosome isolation was performed by a differential ultracentrifugation method.^[^ ^45^ ^]^ Supernatants from the LPS‐activated MΦs cultured on the specimens were collected and centrifuged at 300 ×*g* and 2000 ×*g* for respective 10 min to remove the redundant cells and dead cells, followed by 10 000 ×*g* for 30 min to remove the cell debris. The supernatants were then ultracentrifuged at 100 000 ×*g* for 70 min to pellet exosomes. The exosomes at the bottom of the centrifuge tubes were resuspended in cold phosphate‐buffered saline (PBS), which was further purified by centrifugation at 100 000 ×*g* for another 70 min to eliminate excess proteins. The purified exosomes were resuspended in cold PBS, quantitated with the BCA Protein Assay Kit, and then stored at −80 °C for subsequent experiments. The exosomes from MΦs cultured on the specimens T, T‐A, and T‐AC were, respectively, denoted as T‐Exo, T‐A‐Exo, and T‐AC‐Exo.

Micrographs of the exosomes were recorded by cryo‐transmission electron microscope (Cryo‐TEM, Talos Glacios, Thermo Fisher Scientific, America) and FE‐SEM. The particle size distribution and number of the observed exosomes in FE‐SEM images were analyzed using ImageJ software. The hydrodynamic size of the exosomes was measured by dynamic light scattering (DLS) using a Zetasizer Nano system (ZS90, Malvern, Britain), and the zeta potential of the exosome suspensions was also measured simultaneously. The levels of exosome‐specific markers tumor susceptibility gene 101 protein (TSG101), tetraspanin‐30 (CD63), and tetraspanin‐28 (CD81) were detected by western blotting. The antibodies used were as follows: anti‐TSG101 antibody (sc‐7964, Santa Cruz Biotechnology, America, diluted at 1:1000), anti‐CD63 antibody (sc‐5275, Santa Cruz Biotechnology, America, diluted at 1:1000), anti‐CD81 antibody (sc‐166029, Santa Cruz Biotechnology, America, diluted at 1:1000), anti‐GAPDH antibody (sc‐365062, Santa Cruz Biotechnology, America, diluted at 1:1000), and Goat Anti‐Mouse IgG H&L (HRP) (ab6789, Abcam, Britain, diluted at 1:10000). The proteins extracted from the LPS‐induced MΦs were set as control.

For in vitro experiments, 10 µg mL^−1^ of the isolated exosomes based on protein measurement were given to the recipient cells. The medium was refreshed every 2 days and the exosomes were readded. The groups that cells did not suffer from the exosome stimulation were regarded as controls, named as Control.

### In Vivo Vascular Reconstruction and Bone Regeneration Regulated by the MΦ‐Derived Exosomes

The male SD rats (10 weeks old) were used in this study and anesthetized by intramuscular injections of 1% pentobarbital at a dose of 30 mg kg^−1^ body weight. Subsequently, 1 × 10^6^ ECs were resuspended in 100 µL PBS supplemented with the 100 µg isolated exosomes (only PBS in the control group), followed by mixed with 400 µL of High Concentration Matrigel (Corning, China). The mixtures were then immediately injected subcutaneously into the right lower flanks of the rats. Afterward, the tibial monocortical defect model was established on the tibias of the above rats. The lateral aspects of the rat tibias were fully exposed through longitudinal incisions, and circular defect holes with a diameter of 2 mm were created by surgical electronic drill and carefully rinsed with physiological saline to remove bone dust and fragments. The exosome suspensions (100 µg of the isolated exosomes based on the total protein was suspended in 50 µL PBS) or an equal volume of PBS (50 µL, Control group) were immediately injected near the defects and the injections were performed every 5 days. The control group and each experimental group were set up with 6 rats. Fifteen days after surgery, the Matrigel plugs and defective tibias were excised from the rats.

Newborn bone tissues at monocortical bone defects were detached from the obtained tibias and used to analyze the levels of angiogenesis/osteogenesis markers through western blotting. The antibodies used were as follows: anti‐CD31 antibody (ab281583, Abcam, Britain, diluted at 1:10000), anti‐ANG‐1 antibody (ab183701, Abcam, Britain, diluted at 1:10000), anti‐ALP antibody (sc‐271431, Santa Cruz Biotechnology, America, diluted at 1:1000), anti‐OPN antibody (sc‐21742, Santa Cruz Biotechnology, America, diluted at 1:1000), and secondary antibodies of Goat Anti‐Mouse IgG H&L (HRP) and Goat Anti‐Rabbit IgG H&L (HRP) (ab6721, Abcam, Britain, diluted at 1:10000). GAPDH was used as a housekeeping protein to normalize protein expression. Relative protein expression levels were analyzed semiquantitatively by ImageJ software. The obtained Matrigel plugs were sectioned and stained with hematoxylin and eosin (H&E, Servicebio, China) for histological analysis. The number and lumen area of blood vessels in the sections were measured using ImageJ software. A microcomputed tomography system (Micro‐CT, SkyScan 1176, Bruker, Germany) was performed to observe the new bone formation at bone defects. 3D histomorphometric analysis was implemented on CTAN software using the sagittal images of defective tibias and the bone structure parameters including percent of bone volume/tissue volume (BV/TV), trabecular number (Tb.N), trabecular thickness (Tb.Th), and trabecular separation (Tb.Sp) were measured.

### Exosome Uptake by Recipient Cells

The isolated exosomes were stained by PKH26 Red Fluorescent Cell Linker Kit (Sigma‐Aldrich, American) labeling following the manufacturer's protocols, and the heat‐inactivated 10% BSA solution was used to stop the reaction. The labeled exosomes solution was ultracentrifuged at 100 000 ×*g* for 70 min at 4 °C. Afterward, the exosome pellets were resuspended in cold PBS and applied to incubate recipient cells (ECs and BMSCs) for 2, 4, and 24 h. At each time point, the cells were counterstained with FITC and DAPI and then observed by the CLSM. Semiquantitative MFI of the labeled exosomes taken up by a single cell at each time point was analyzed by ImageJ software, while the area and perimeter of the cell cytoskeleton were calculated by the software.

### Cell Adhesion, Viability, Proliferation, and Morphology of the Recipient Cells Regulated by the MΦ‐Derived Exosomes

Cell adhesion, viability, proliferation, and morphology were assessed following the methods described in the previous study.^[^ ^4^ ^]^ The cells were seeded on chamber slides in 24‐well cell culture plates and the exosomes were immediately added into the wells. After 0.5, 1, and 4 h of incubation, cells were fixed in 4% PFA, stained with DAPI, and photographed by the CLSM. The number of cell nuclei was calculated using ImageJ software. Cell viability and proliferation were assessed using Live/Dead fluorescence staining and MTT assay after 2, 4, and 6 days of the exosome stimulation. Cell morphology was observed by an optical microscope (ECLIPSE Ts2R‐FL, Nikon, Japan) and FE‐SEM after being stimulated by the exosomes for 12 and 24 h. The cells in optical micrographs were pseudocolored using Adobe Photoshop software. The spreading area and perimeter of the cells in FE‐SEM images were analyzed by ImageJ software.

### In Vitro Angiogenesis of ECs Regulated by the MΦ‐Derived Exosomes

After ECs were stimulated by the exosomes for 2 days, immunofluorescence staining assay was applied to evaluate CD31 synthesis. Anti‐CD31 antibody (diluted at 1:1000) and a secondary antibody of Goat Anti‐Rabbit IgG H&L (Alexa Fluor 488) were used in the assay. Cell nuclei were counterstained with DAPI and fluorescent images were captured using the CLSM. Semiquantitative MFI of labeled CD31 per cell was measured by ImageJ software. Wound‐healing assay was used to evaluate the cell migration.^[^ ^4^ ^]^ After cultured for 3 days to reach ≈90% confluence, monolayer ECs were scratched with a 200 µL pipette tip and rinsed thrice with sterile PBS to remove the cell debris. Afterward, ECs were incubated in serum‐free DMEM supplemented with different exosomes. At time points of 0, 10, 20, and 30 h after the scratch forming, images of monitoring EC migration were taken using the optical microscope, and the cell migration rate was calculated. The in vitro angiogenesis assay was conducted using ECMatrix (ECM625, Millipore, America) according to the manufacturer's specifications. The ECs resuspended in the medium containing the exosomes were seeded on ECMatrix gel at the density of 6 × 10^4^ cells per well and incubated for 4, 8, 12, and 18 h. At each time point, the images from at least six random fields were photographed using the optical microscope. The number of branch points in cell lines (nodes), mesh‐like circles (circles), and tube‐like parallel cell lines (tubes) were calculated using ImageJ software.

### In Vitro Osteogenic Differentiation of BMSCs Regulated by the MΦ‐Derived Exosomes

For osteogenic differentiation induction, the seeded BMSCs were cultured normally for 3 days and then the medium was substituted by the exosome‐deprived osteogenic induction medium (the α‐MEM supplemented with 10 mm β‐glycerophosphate, 50 µg mL^−1^ ascorbic acid, and 100 nm dexamethasone), while the exosomes were given to BMSCs. Osteogenic differentiation of BMSCs was assessed by alkaline phosphatase (ALP) activity, collagen secretion, and extracellular matrix (ECM) mineralization as described in the previous study.^[^ ^4^ ^]^ After 3 and 7 days of osteogenic differentiation induction and exosome stimulation, ALP activity was assayed qualitatively using BCIP/NBT Alkaline Phosphate Color Development Kit (Beyotime, China) and quantitatively by Alkaline Phosphatase Assay Kit (Beyotime, China) according to the manufacturer's instructions. After induction for 7 and 14 days, collagen secretion was evaluated by Sirius Red staining. After 14 and 21 days of induction, ECM mineralization was assessed by Alizarin Red S staining.

### Internalization, Trafficking, and Degradation of Integrin β1 Mediated by the MΦ‐Derived Exosomes

Immunofluorescence staining was adopted to detect integrin β1 (ITGβ1) internalization of the recipient cells. After the seeded recipient cells were stimulated with the exosomes for 2 and 6 h, the cells were fixed with 4% PFA, permeabilized with 0.1% saponin, blocked with 1% BSA, and incubated with mouse monoclonal anti‐ITGβ1 antibody (sc‐53711, Santa Cruz Biotechnology, America, diluted at 1:200) overnight at 4 °C. The secondary antibody Goat Anti‐Mouse IgG H&L (Alexa Fluor 555) (ab150114, Abcam, Britain, diluted at 1:1000) was used to incubate cells at room temperature for 1 h to couple primary antibodies. Afterward, the cytoskeleton and nuclei were counterstained with FITC and DAPI, respectively. The permeabilization step was elided to detect ITGβ1 on the cell membranes. Fluorescent images were captured using the CLSM and semiquantitative analysis of MFI for labeled ITGβ1 per cell was analyzed by ImageJ software. After the recipient cells were incubated with the exosomes for 6 h, total ITGβ1 levels in the cells were analyzed by enzyme‐linked immunosorbent assay (ELISA) and western blotting. ITGβ1 ELISA Kit (mlbio, China) was applied to quantify cellular ITGβ1 levels following the manufacturer's instructions. In addition, anti‐ITGβ1 antibody (diluted at 1:1000) and secondary antibody of Goat Anti‐Mouse IgG H&L (HRP) were applied in western blotting. To suppress the lysosomal degradation function, bafilomycin A (20 ng mL^−1^) was adopted to treat cells for 3 h. Cell morphology was captured by the optical microscope. Colocalization analysis of ITGβ1 and lysosome was performed by immunofluorescence staining assay. The primary antibodies of ITGβ1 and lysosomal‐associated membrane protein 1 (LAMP1) (ab24170, Abcam, Britain, diluted at 1:1000) were used to incubate the cells overnight at 4 °C. The secondary antibodies of Goat Anti‐Mouse IgG H&L (Alexa Fluor 488) and Goat Anti‐Rabbit IgG H&L (Alexa Fluor 555) were applied to incubate the cells to visualize the ITGβ1 and lysosomes, respectively. Cell nuclei were stained with DAPI and fluorescent images were captured by the CLSM.

### Transient Transfection of ITGβ1

Small interfering RNA (siRNA) transfection was applied to silence ITGβ1 expression in ECs. ITGβ1 siRNA duplex (sc‐35674, Santa Cruz Biotechnology, America) and siRNA transfection reagent (sc‐29528, Santa Cruz Biotechnology, America) were used to transfect ECs according to the manufacturer's guidelines. Negative control siRNA (sc‐37007, Santa Cruz Biotechnology, America) was used for control transfection. The ITGβ1‐knockdown (KD) (ITGβ1*^KD^*) ECs were cultured for an additional 24, 48, and 72 h and the cell morphology was captured on the optical microscope. Transfection efficiency was evaluated by reverse transcription‐quantitative real‐time polymerase chain reaction (RT‐qPCR) after transfection for 24 h and western blotting after transfection for 72 h. Target primer sequences used in RT‐qPCR are listed as follows: human ITGβ1 (forward: 5′‐AGATGTGTCAGACCTGCCTTGG‐3′; reverse: 5′‐AATTTGTCCCGACTTTCTACCTTGG‐3′) and human GAPDH (forward: 5′‐CAGGAGGCATTGCTGATGAT‐3′; reverse: 5′‐GAAGGCTGGGGCTCATTT‐3′). Cell migration and in vitro angiogenesis of ITGβ1*^KD^* ECs were assessed using the wound‐healing model and ECMatrix gel as described previously. The ECs transfected by negative control siRNA were set as control.

### Colocalization Analysis of the Internalized ITGβ1 and Exosomes

The isolated exosomes were labeled with the red fluorescent dye PKH26 and used to incubate the recipient cells for 1, 2, and 4 h. The cells were rinsed with PBS, fixed with 4% PFA, permeabilized with 0.1% saponin, blocked with 1% BSA, and incubated with anti‐ITGβ1 antibody. A secondary antibody Goat Anti‐Mouse IgG H&L (Alexa Fluor 488) (ab150113, Abcam, Britain, diluted at 1:1000) was applied to visualize the internalized ITGβ1. Cell nuclei were stained with DAPI and the fluorescent images were captured by the CLSM.

### Proteinase K Treatment of the Exosomes

The exosomes were treated with 10 µg mL^−1^ of Proteinase K (Sangon Biotech, China) for 10 min at 37 °C to degrade outer membrane proteins and the procedure was terminated with an excess of heat‐inactivated 10% BSA solution. The exosomes were recollected by ultracentrifugation and then used to stimulate the recipient cells. In this experiment, T‐Exo was chosen for the assay and the cell groups stimulated with Proteinase K‐treated exosomes (PKT‐Exo) were the experimental group. The groups not stimulated by the exosomes (Control) and the groups stimulated by normal exosomes (N‐Exo) were used as control groups. The exosome internalization was detected by fluorescence staining, total ITGβ1 levels were analyzed by western blotting, and cell morphology was observed by optical microscope.

### Cytokine Array Analysis

Proteome Profiler Mouse Cytokine Array (ARY006, R&D Systems, America) was applied to detect the exosome‐bound cytokines according to the manufacturer's instructions. Two hundred micrograms of the exosomes based on total protein content were applied for one test (the exosomes were enriched to a concentration of 200 µg mL^−1^ and 1 mL of exosome suspension was used in the assay). Semiquantitative integrated gray‐scale density of array spots was measured using ImageJ software.

### ELISA Tests of Exosome‐Bound GM‐CSF, CCL2, and CXCL2

The exosome suspensions were normalized to a protein concentration of 200 µg mL^−1^ and applied to quantify the levels of exosome‐bound GM‐CSF using a Mouse GM‐CSF ELISA Kit (mlbio, China) following the instructions. Additionally, levels of exosome‐bound CCL2 and CXCL2 were analyzed, respectively, using Mouse CCL2 ELISA Kit (mlbio, China) and Mouse CXCL2 ELISA Kit (mlbio, China). The quantitative results of cytokine levels were converted into actual action levels of the exosomes at 10 µg mL^−1^ protein concentration according to the concentration ratio. PKT‐Exo‐bound GM‐CSF, CCL2, and CXCL2 were also detected by ELISA.

### Gene Expression and Cytokine Secretion of the Specimen‐Mediated MΦs

Differential gene expression was analyzed through mRNA sequencing on a sequencing platform MGISEQ‐2000 (BGI, China). The expression levels of genes associated with the cytokines screened by the cytokine array and involved in major signaling pathways were obtained from the differential gene profiles. Gene expression levels were presented as relative values of FPKM (fold change). Cytokines GM‐CSF, CCL2, and CXCL2 in MΦ supernatants were detected by ELISA.

### Concentration‐Dependent Effects of GM‐CSF, CCL2, and CXCL2 on ITGβ1 Levels

The recipient cells were incubated for 6 h in serum‐free medium containing 0, 12.5, 25, 50, 100, 200, and 400 ng mL^−1^ of recombinant murine GM‐CSF (Sangon Biotech, China). Subsequently, the cells were imaged by the optical microscope, and the total ITGβ1 levels were detected by ELISA. Recombinant murine CCL2 and CXCL2 (Sangon Biotech, China) were used to stimulate the recipient cells to evaluate their effects on ITGβ1 levels as mentioned above.

### Antagonism of CCR2 Binding Activity

INCB3344 (HY‐50674, MCE, America) was adopted to antagonize the binding activity of CCR2, whereby ECs were incubated with 10 nm INCB3344 for 30 min and BMSCs were incubated with 15 nm INCB3344 for 30 min. Subsequently, 50 ng mL^−1^ CCL2 or 10 µg mL^−1^ exosomes (T‐Exo) were used to incubate the cells for 6 h, followed by the observation of cell morphology by optical microscope and analysis of ITGβ1 levels by western blotting. The cells with antagonistic CCR2 binding were indicated as CCR2^−^ and the cells with normal CCR2 binding activity were denoted as CCR2^+^. The cells stimulated by CCL2 (or exosomes) were denoted as CCL2^+^ (or Exo^+^), otherwise as CCL2^−^ (or Exo^−^).

### Cellular Autophagy Assay

The recipient cells were stimulated with 50 ng mL^−1^ CCL2 and 10 µg mL^−1^ exosomes for 4 h, followed by stained with Autophagy Staining Assay Kit with Monodansylcadaverine (Beyotime, China) and counterstained with Hoechst 33 342 (Beyotime, China). The cells were observed on the CLSM and the levels of fluorescently labeled autophagosomes were semiquantitatively analyzed using ImageJ software.

### Statistical Analysis

Statistical analysis was conducted using SPSS software (v14.0, IBM, America). All data were presented as means ± standard deviation (SD). A two‐tailed unpaired Student's *t*‐test was applied to compare the data between two‐group, while a one‐way analysis of variance (ANOVA) with the Student‐Newman–Keuls (SNK) post hoc test was applied to analyze the data among multiple‐groups. The sample size (n) was shown in the corresponding figure legends. Values of *p* < 0.05, *p* < 0.01, and *p* < 0.001 were considered to be statistically significant, highly significant, and extremely highly significant, respectively.

## Conflict of Interest

The authors declare no conflict of interest.

## Supporting information



Supporting Information

## Data Availability

The data that support the findings of this study are available in the Supporting Information of this article.
