# Supplementary material for: Biomaterial Surface‐Mediated Macrophages Exert Immunomodulatory Roles by Exosomal CCL2‐Induced Membrane Integrin β1 Trafficking in Recipient Cells
Source: Adv Sci (Weinh). 2025 Jan 21;12(10):2409809. doi: 10.1002/advs.202409809 (PMC11905086; doi:10.1002/advs.202409809)
Supplement: Supplementary file 1 — Supporting Information [file ADVS-12-2409809-s001.docx]

Supplementary Information

**Biomaterial Surface-Mediated Macrophages Exert Immunomodulatory Roles by Exosomal CCL2-Induced Membrane Integrin β1 Trafficking in Recipient Cells**

*Yuyu Zhao^1^, Ruiyue Hang^1^, Huifei Li^2^, Yonghua Sun^1^, Runhua Yao^1^, Xiaobo Huang^1^, Xiangyu Zhang^1^, Xiaohong Yao^1,^ *, Huaiyu Wang^4^, Yin Xiao^5^, Di Huang^6^, Yong Han^3,^ *, Xing Wang^2,^ *, Ruiqiang Hang^1, 3,^ **

*^1^* *Shanxi Key Laboratory of Biomedical Metal Materials, College of Materials Science and Engineering, Taiyuan University of Technology, Taiyuan 030024, China*

*^2^ School and Hospital of Stomatology, Shanxi Medical University, Taiyuan 030001, China*

*^3^ State-Key Laboratory for Mechanical Behavior of Materials, Xi'an Jiaotong University, Xi'an, 710049 China*

*^4^ Center for Human Tissues and Organs Degeneration, Shenzhen Institute of Advanced Technology, Chinese Academy of Sciences, Shenzhen 518055, China*

*^5^ School of Medicine and Dentistry, Griffith University, Gold Coast, QLD 4222, Australia*

*^6^ Research Center for Nano-Biomaterials & Regenerative Medicine, Department of Biomedical Engineering, College of Biomedical Engineering, Taiyuan University of Technology, Taiyuan, 030024 China*

* Correspondence: hangruiqiang@tyut.edu.cn (R.Q. Hang); wangxing@sxmu.edu.cn (X. Wang); yonghan@mail.xjtu.edu.cn (Y. Han); xhyao@tyut.edu.cn (X.H. Yao)


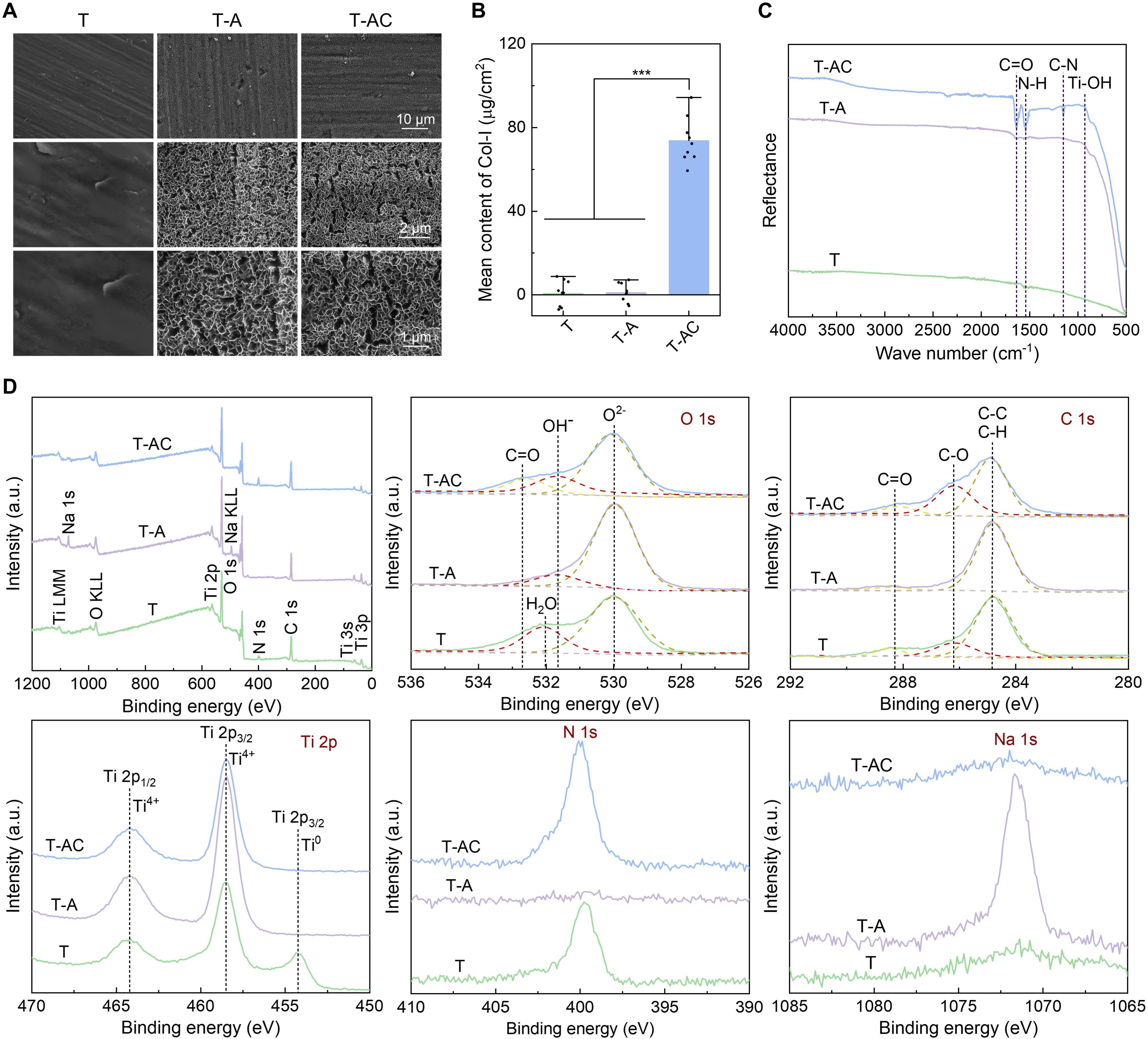


**Figure S1.** Surface characterization of the specimens. A) Surface topographies of the specimens observed by field-emission scanning electron microscope (FE-SEM). B) Immobilized Col-I content on the specimen surfaces (n = 9). C) Surface chemical components of the specimens detected by diffuse reflectance Fourier transform infrared spectroscope (DR-FTIR). D) Surface element composition of the specimens tested by X-ray photoelectron spectroscope (XPS) (survey spectra and high-resolution spectra of O 1s, C 1s, Ti 2p, N 1s, and Na 2p). T: untreated pure titanium (Ti), T-A: nanostructured Ti, and T-AC: type I collagen (Col-I)-decorated nanostructured Ti. Data are presented as means ± SD. ^***^*p* < 0.001.


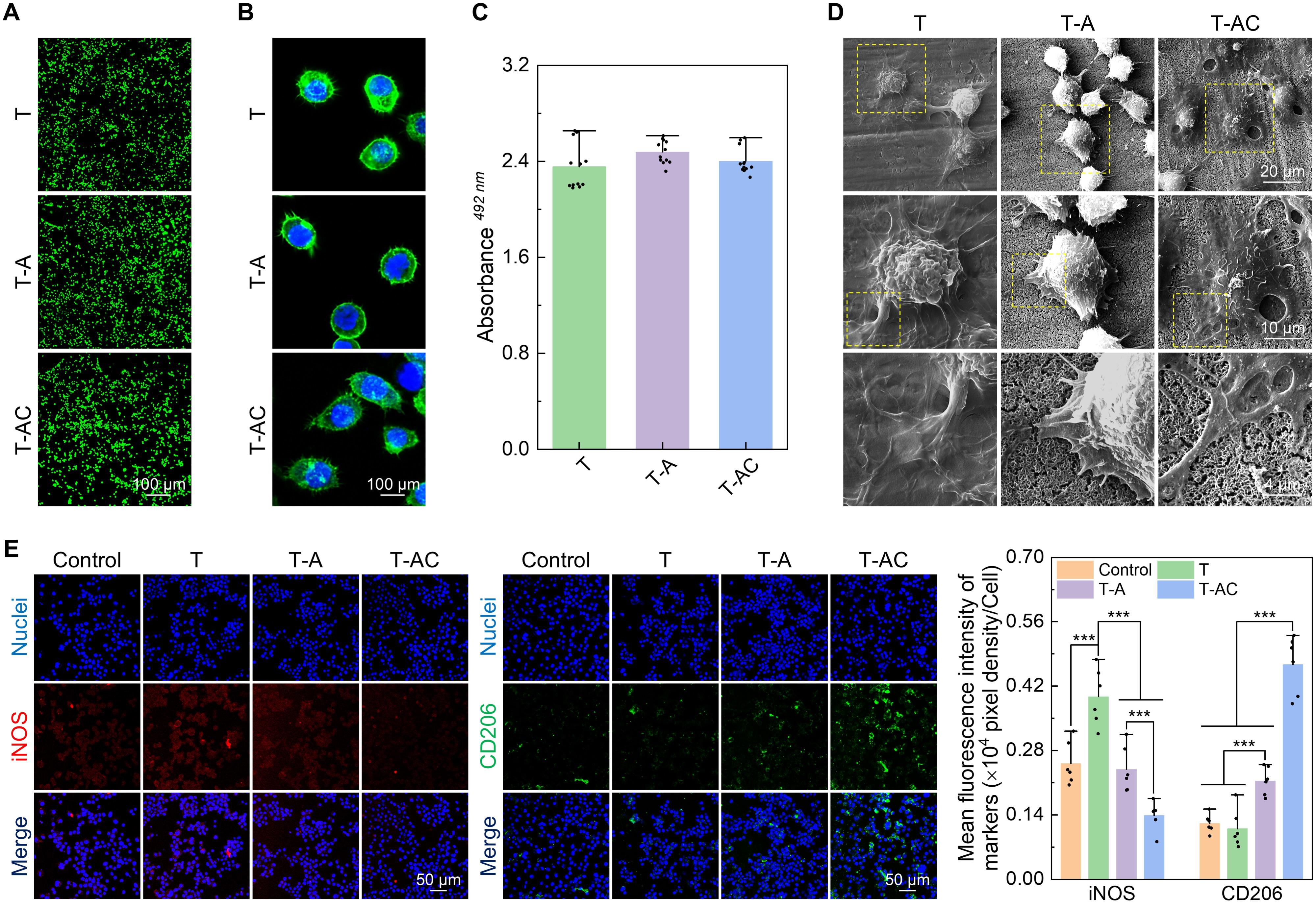


**Figure S2.** Cell viability and phenotype polarization of macrophages (MΦs) mediated by the specimens. A) Fluorescence images of Live/Dead staining for MΦ viability. B) Fluorescence images for MΦ cytoskeleton assembly. C) Assessment for MΦ proliferation determined by MTT assay (n = 12). D) FE-SEM images of MΦ morphology. E) Immunofluorescence staining images for the M1/M2 phenotypic markers (inducible nitric oxide synthase (iNOS)/MΦ mannose receptor C-type 1 (CD206)) of MΦs and quantitative analysis (n = 6). Data are presented as means ± SD. ^***^*p* < 0.001.


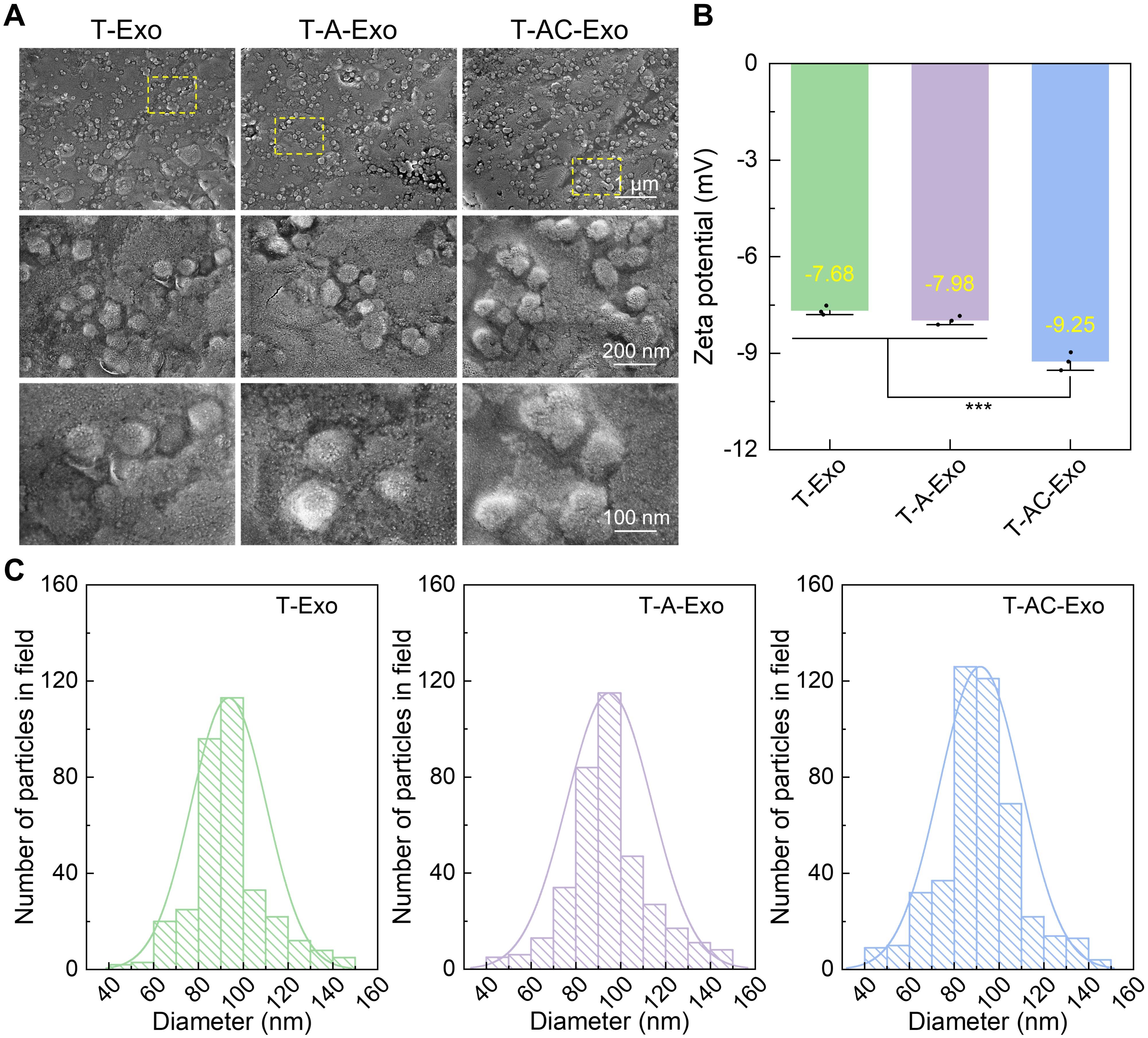


**Figure S3.** Characterization of the MΦ-derived exosomes. A) Representative FE-SEM micrographs of the exosomes. B) Zeta potential of the exosome suspensions (n = 3). C) Size distribution of the exosomes in FE-SEM images. Data are presented as means ± SD. ^***^*p* < 0.001.


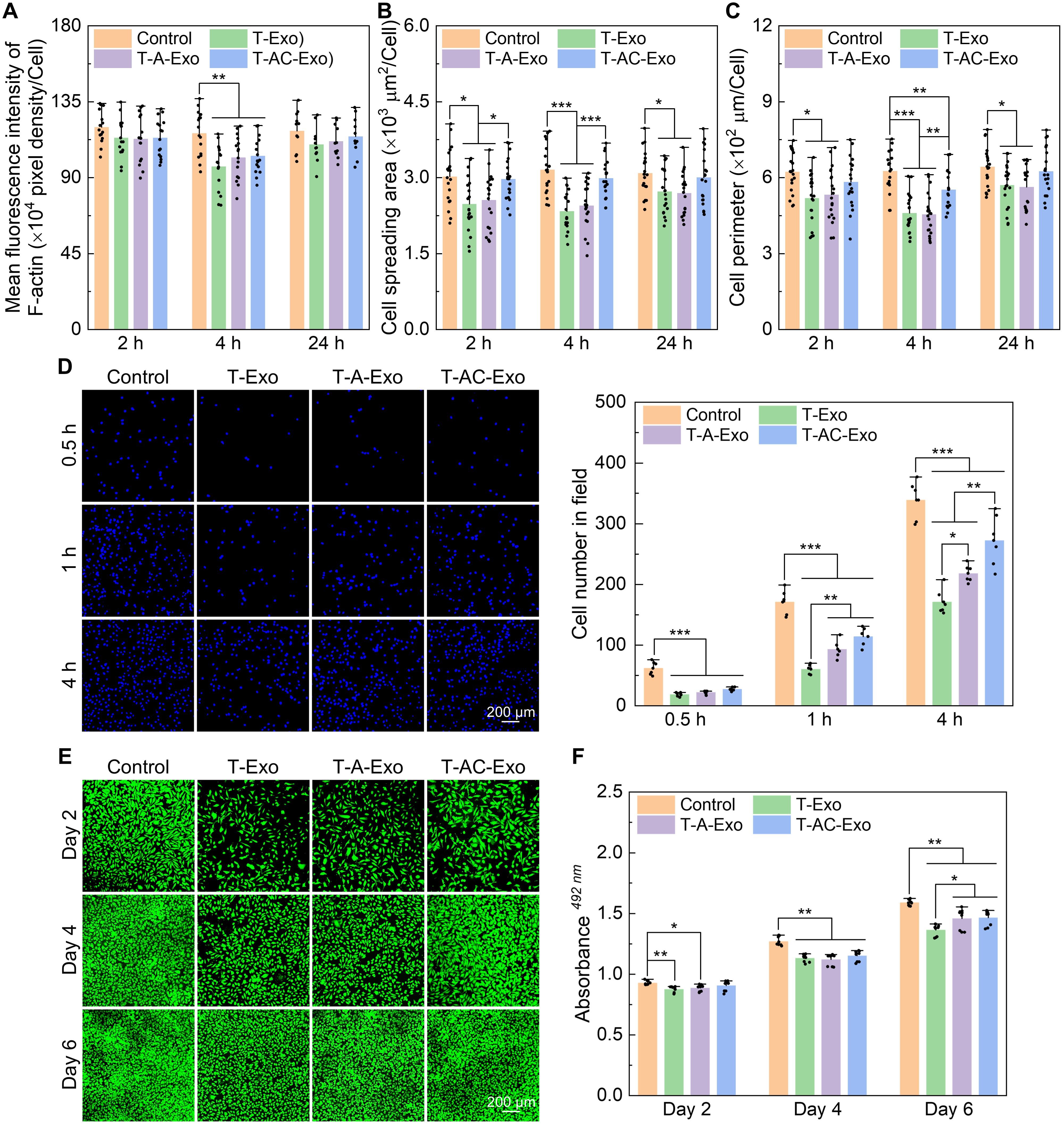


**Figure S4.** Distinct in-vitro cell viability and behaviors of endothelial cells (ECs) regulated by the exosomes derived from MΦs cultured on different specimen surfaces. A) Quantitative analysis of F-actin fluorescence intensity (n = 15). B) Quantitative analysis of cellular spreading area presented by the cytoskeleton (n = 20). C) Quantitative analysis of cellular perimeter presented by the cytoskeleton (n = 20). D) Fluorescence staining images and quantitative statistics for nuclei of initial adherent cells (n = 6). E) Fluorescence images of Live/Dead staining for cell viability. F) Assessment for cell proliferation determined by MTT assay (n = 9). Data are presented as means ± SD. ^*^*p* < 0.05, ^**^*p* < 0.01, and ^***^*p* < 0.001.


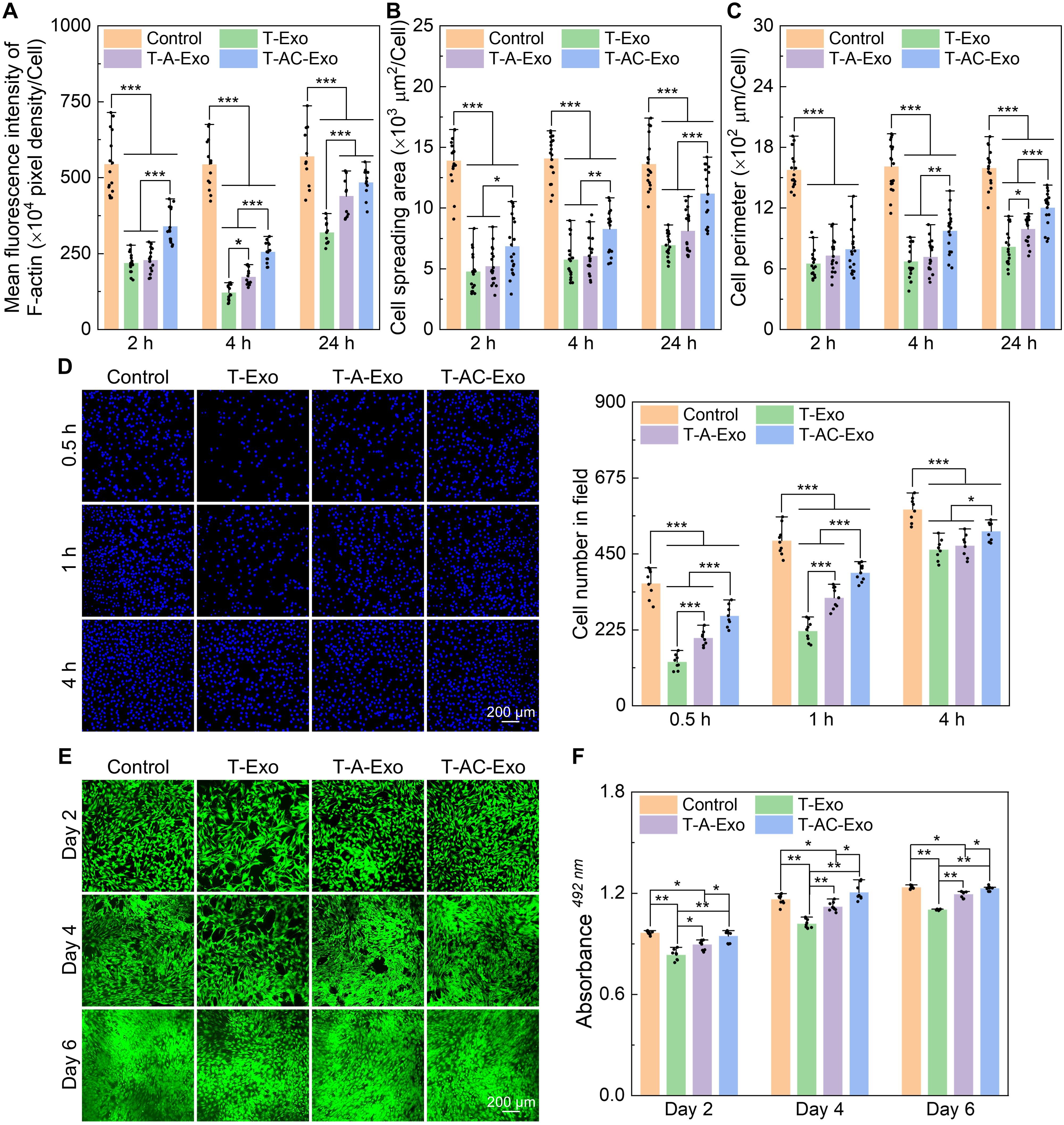


**Figure S5.** Distinct in-vitro cell viability and behaviors of bone marrow mesenchyml stem cells (BMSCs) regulated by the exosomes derived from MΦs cultured on different specimen surfaces. A) Quantitative analysis of F-actin fluorescence intensity (n = 11). B) Quantitative analysis of cellular spreading area presented by the cytoskeleton (n = 18). C) Quantitative analysis of cellular perimeter presented by the cytoskeleton (n = 18). D) Fluorescence staining images and quantitative statistics for nuclei of initial adherent cells (n = 8). E) Fluorescence images of Live/Dead staining for cell viability. F) Assessment for cell proliferation determined by MTT assay (n = 9). Data are presented as means ± SD. ^*^*p* < 0.05, ^**^*p* < 0.01, and ^***^*p* < 0.001.


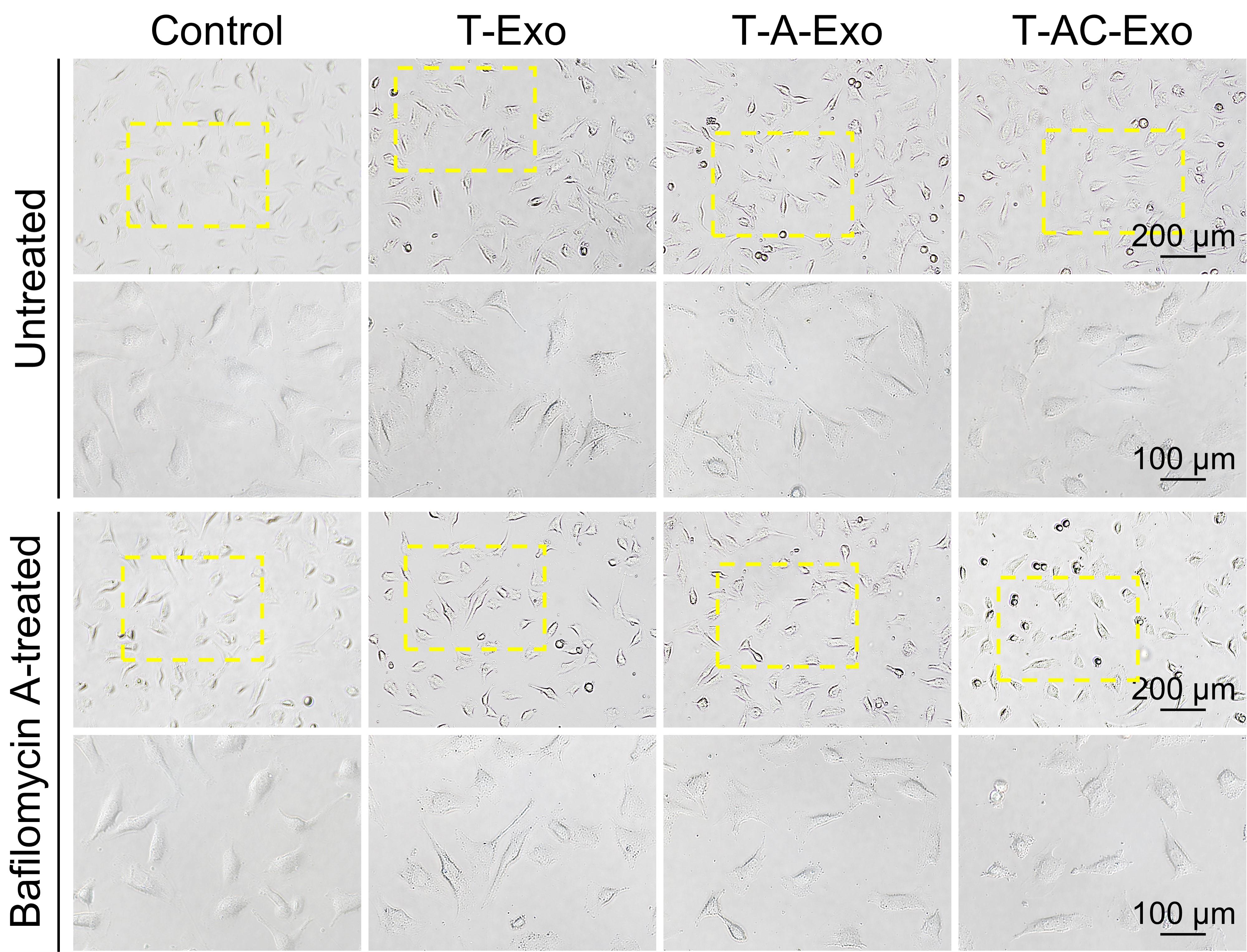


**Figure S6.** Morphology of ECs regulated by the exosomes derived from MΦs cultured on different specimen surfaces. Optical images were captured after 6 h of the exosome incubation, and bafilomycin A was used to inhibit lysosomal degradation.


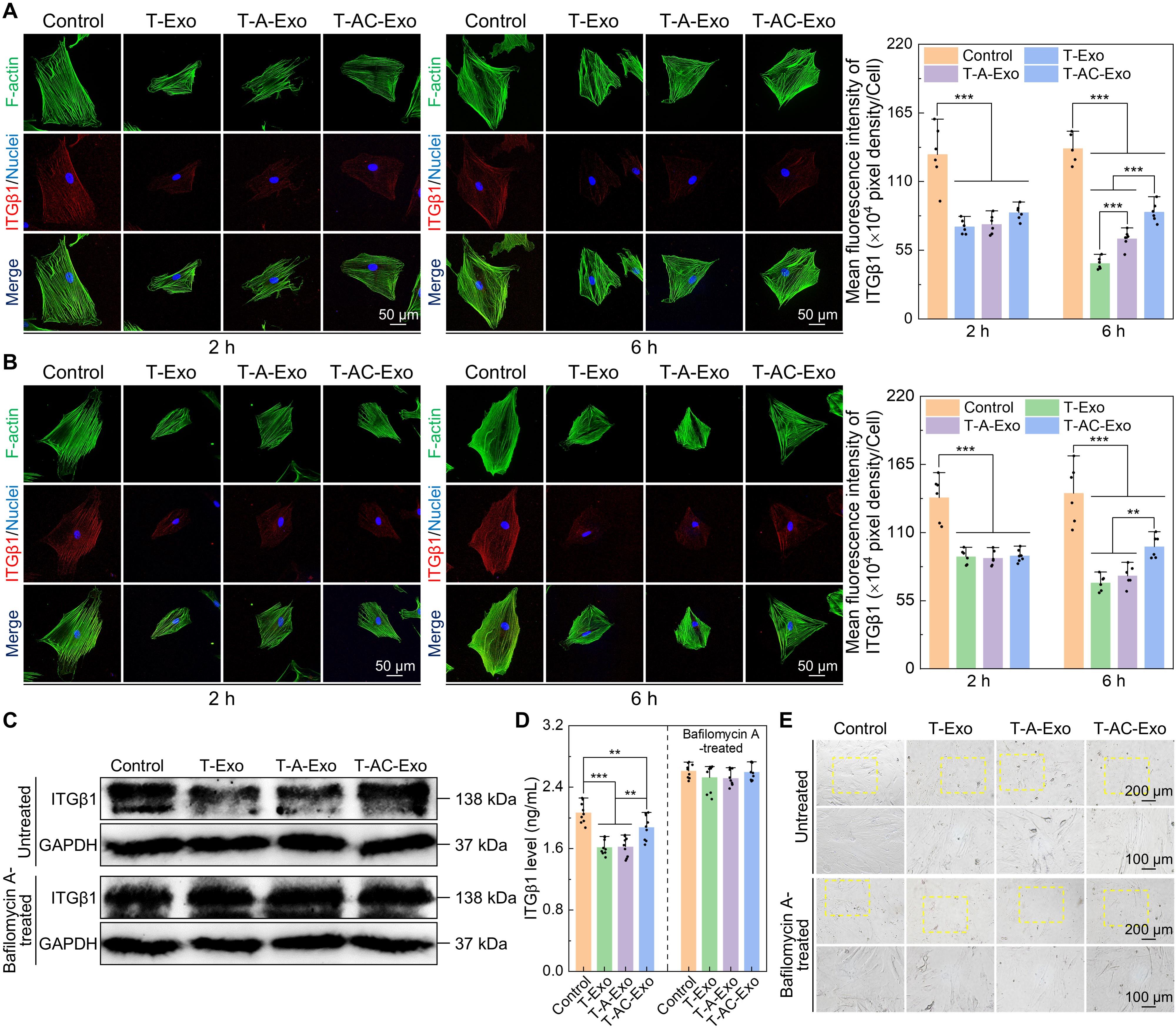


**Figure S7.** Internalization and degradation of membrane integrin β1 (ITGβ1) of BMSCs distinctively regulated by the exosomes derived from MΦs cultured on different specimen surfaces. A) Immunofluorescence staining images of membrane ITGβ1 (omitted cell permeabilization) and quantitative analysis (n = 6). B) Immunofluorescence staining images of total ITGβ1 and quantitative analysis (n = 6). C) Western blotting analysis for cellular total ITGβ1 levels after incubated with the exosomes for 6 h (bafilomycin A was used to inhibit lysosomal degradation). D) Enzyme-linked immunosorbent assay (ELISA) for cellular total ITGβ1 levels after incubated with the exosomes for 6 h (n = 9). E) Cellular optical morphology after incubated with the exosomes for 6 h. Data are presented as means ± SD. ^**^*p* < 0.01 and ^***^*p* < 0.001.


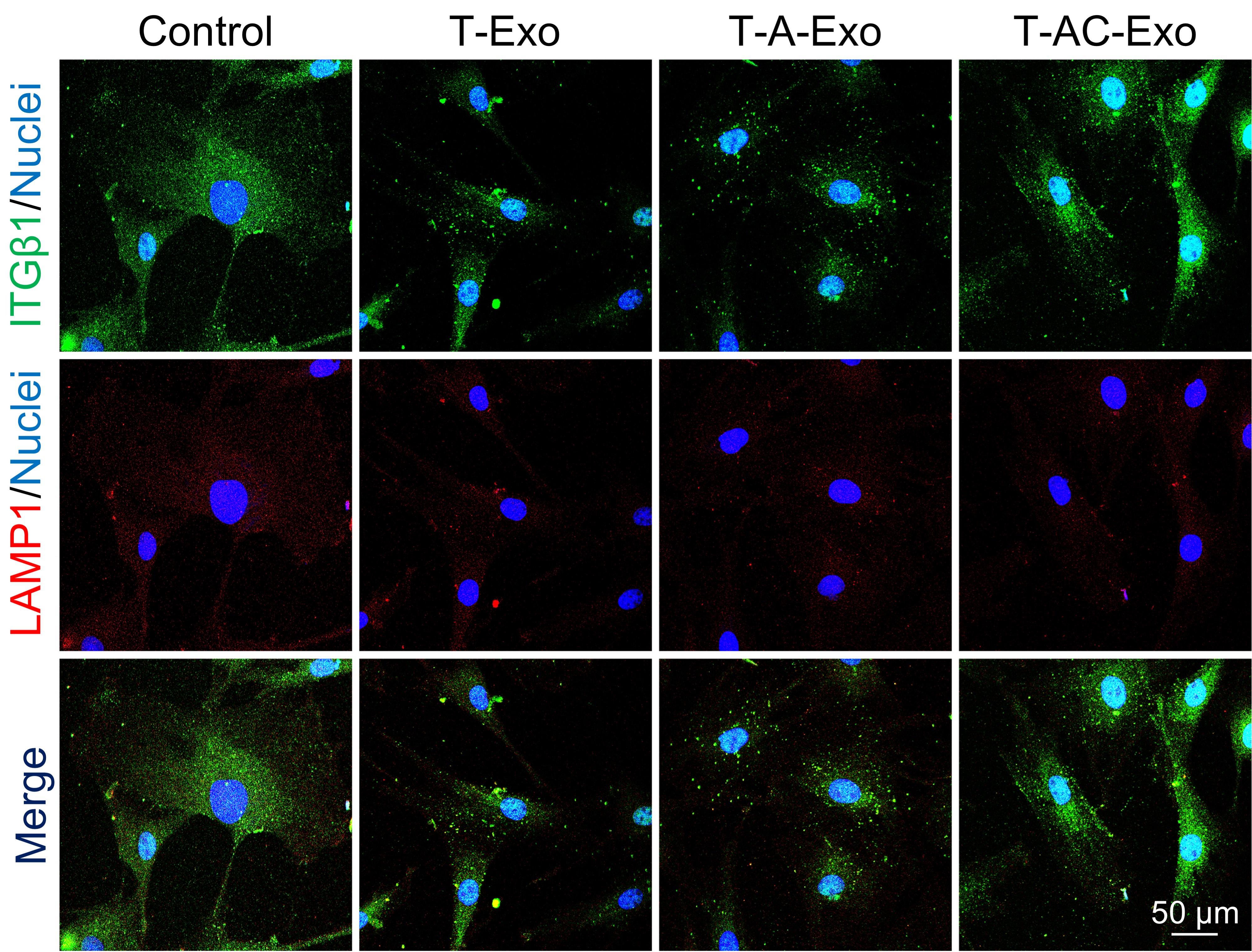


**Figure S8.** Colocalization analysis of ITGβ1 with lysosomal-associated membrane protein 1 (LAMP1) in BMSCs. Fluorescence images were captured after incubated with the MΦ-derived exosomes for 4 h.


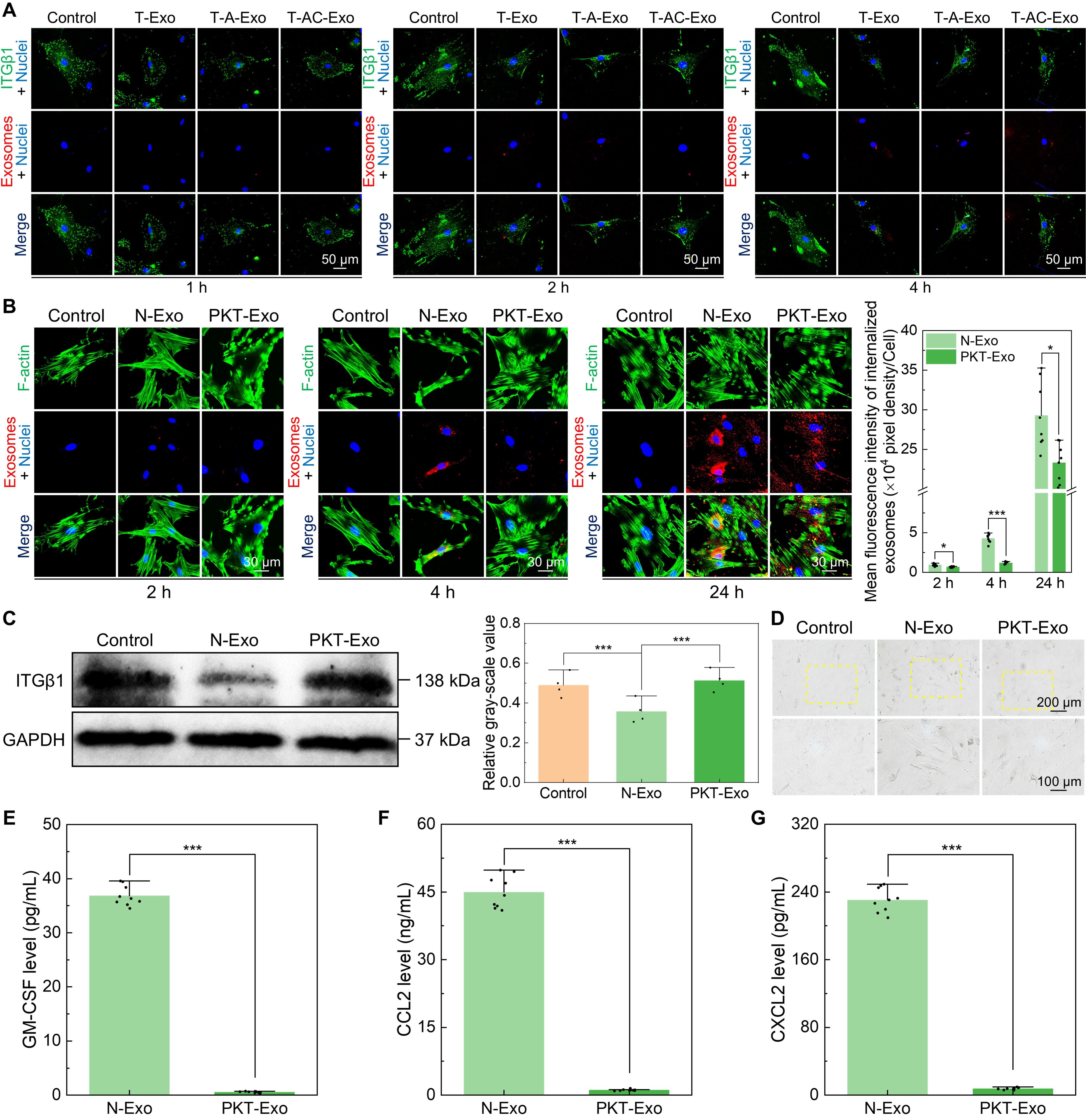


**Figure S9.** Determination of key regulatory components of the MΦ-derived exosomes. A) Colocalization analysis of ITGβ1 with internalized exosomes in BMSCs. B) Exosome internalization detection of normal exosomes (N-Exo) and Proteinase K-treated exosomes (PKT-Exo) in BMSCs (fluorescence images and quantitative analysis of the exosomes taken up by cells) (n = 8). C) Western blotting analysis for total ITGβ1 levels in BMSCs mediated by N-Exo and PKT-Exo after 6 h of incubation (protein blotting images and quantitative analysis) (n = 4). D) Cell morphology of BMSCs mediated by N-Exo and PKT-Exo after 6 h of incubation. E-G) Quantification for levels of the exosome-bound granulocyte-MΦ colony-stimulating factor (GM-CSF), C-C motif chemokine ligand 2 (CCL2), and C-X-C motif chemokine ligand 2 (CXCL2) of N-Exo and PKT-Exo determined by ELISA (n = 9). Data are presented as means ± SD. ^*^*p* < 0.05 and ^***^*p* < 0.001.


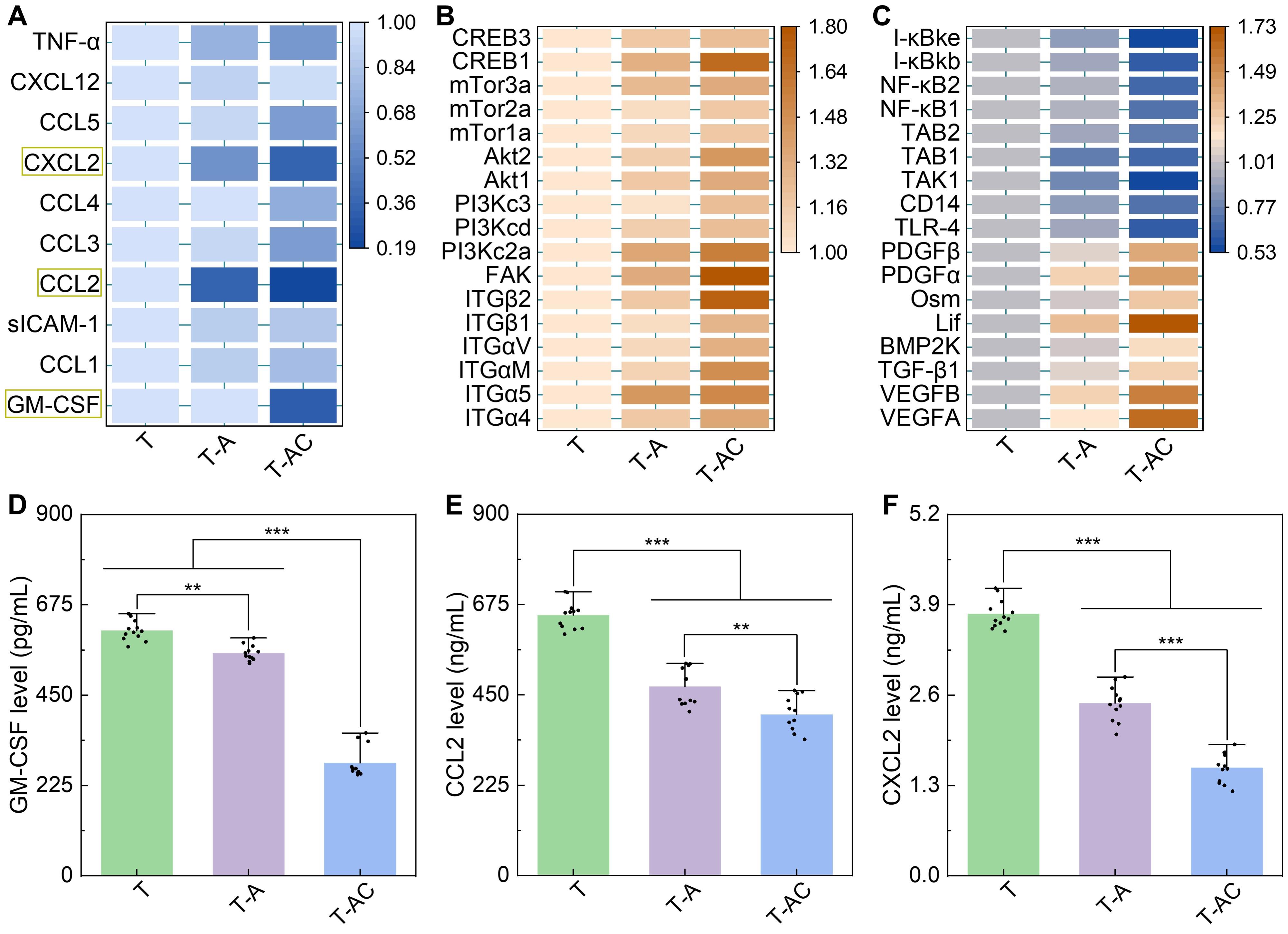


**Figure S10.** Gene expression and cytokine secretion of MΦs mediated by different specimen surfaces. A-C) Gene expression of cytokines and key signaling pathways tested by mRNA sequencing (n = 3). D-F) The levels of GM-CSF, CCL2, and CXCL2 in MΦ supernatants detected by ELISA (n = 12). Data are presented as means ± SD. ^**^*p* < 0.01 and ^***^*p* < 0.001.


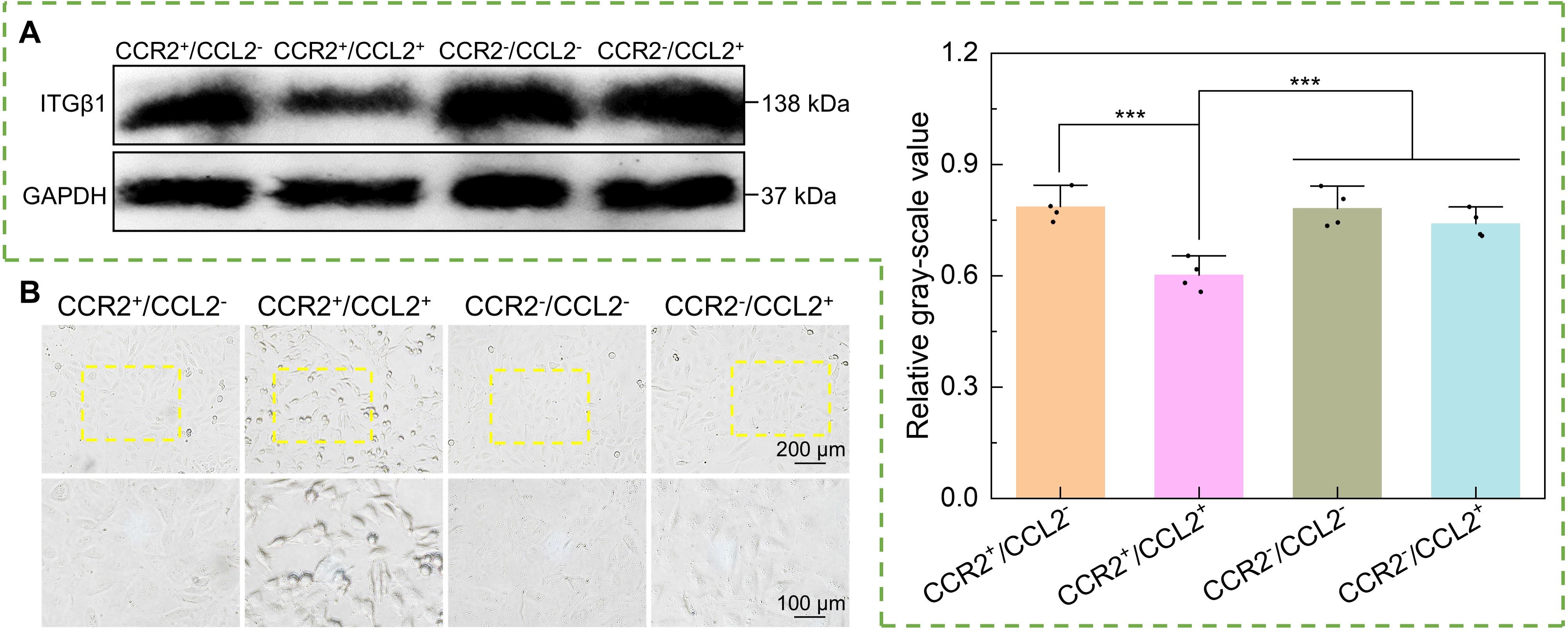


**Figure S11.** ITGβ1 levels and cell morphology of ECs regulated by CCL2. A) Western blotting analysis for total ITGβ1 levels mediated by CCL2/C-C motif chemokine receptor 2 (CCR2) binding after stimulated with CCL2 for 6 h (n = 4). B) Cell morphology mediated by CCL2/CCR2 binding after stimulated with CCL2 for 6 h. Data are presented as means ± SD. ^***^*p* < 0.001.


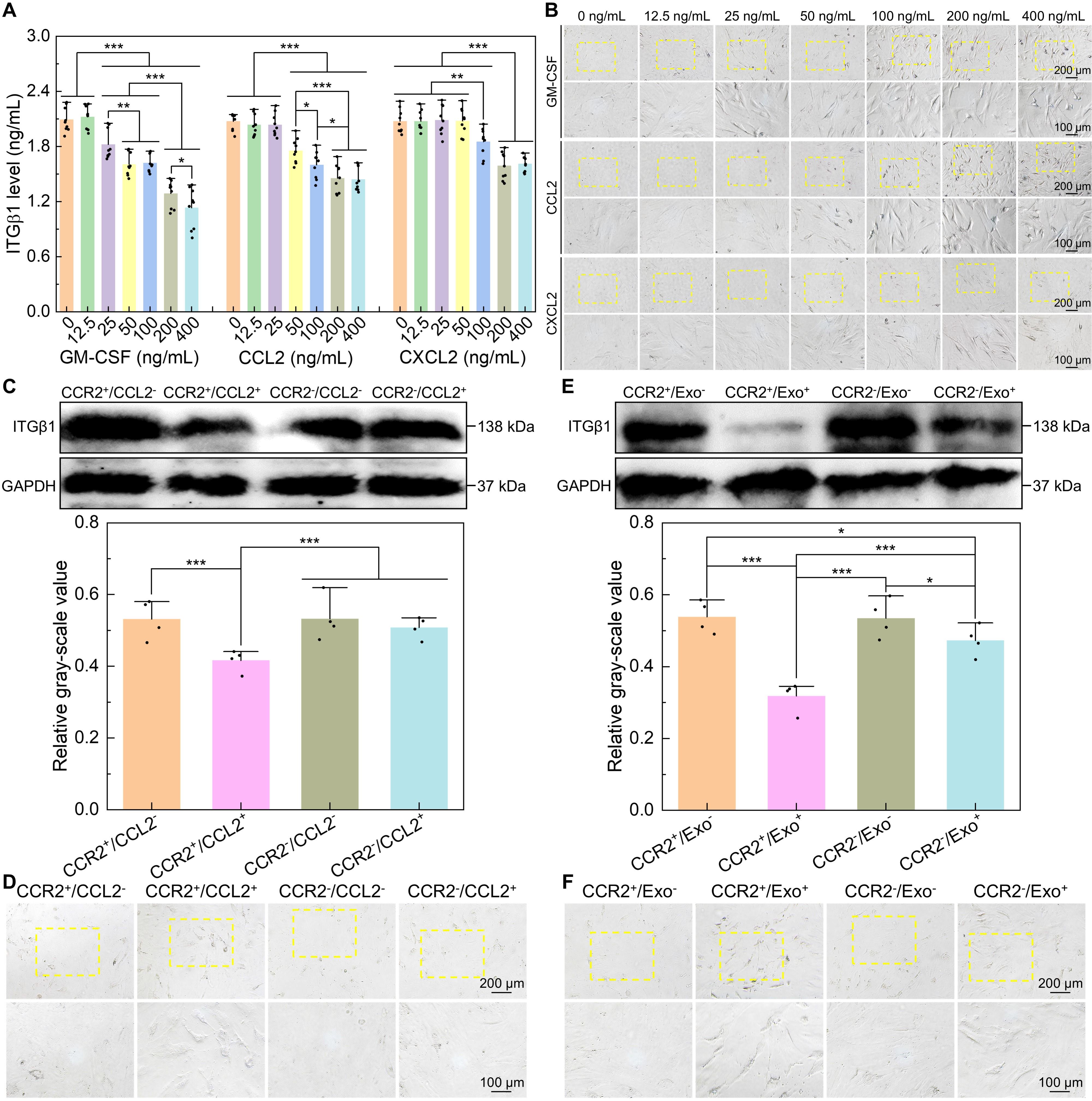


**Figure S12.** ITGβ1 levels and cell morphology of BMSCs regulated by CCL2 and exosome. A) ELISA test for cellular total ITGβ1 levels after stimulated with gradient concentrations of GM-CSF, CCL2, and CXCL2 for 6 h (n = 9). B) Cell morphology after stimulated with GM-CSF, CCL2, and CXCL2 for 6 h. C) Western blotting analysis for total ITGβ1 levels mediated by CCL2/CCR2 binding after stimulated with CCL2 for 6 h (protein blotting images and quantitative analysis) (n = 4). D) Cell morphology mediated by CCL2/CCR2 binding after stimulated with CCL2 for 6 h. E) Western blotting analysis for total ITGβ1 levels mediated by exosome/CCR2 binding after incubated with the exosome for 6 h (protein blotting images and quantitative analysis) (n = 4). F) Cell morphology mediated by exosome/CCR2 binding after incubated with the exosome for 6 h. Data are presented as means ± SD. ^*^*p* < 0.05, ^**^*p* < 0.01, and ^***^*p* < 0.001.


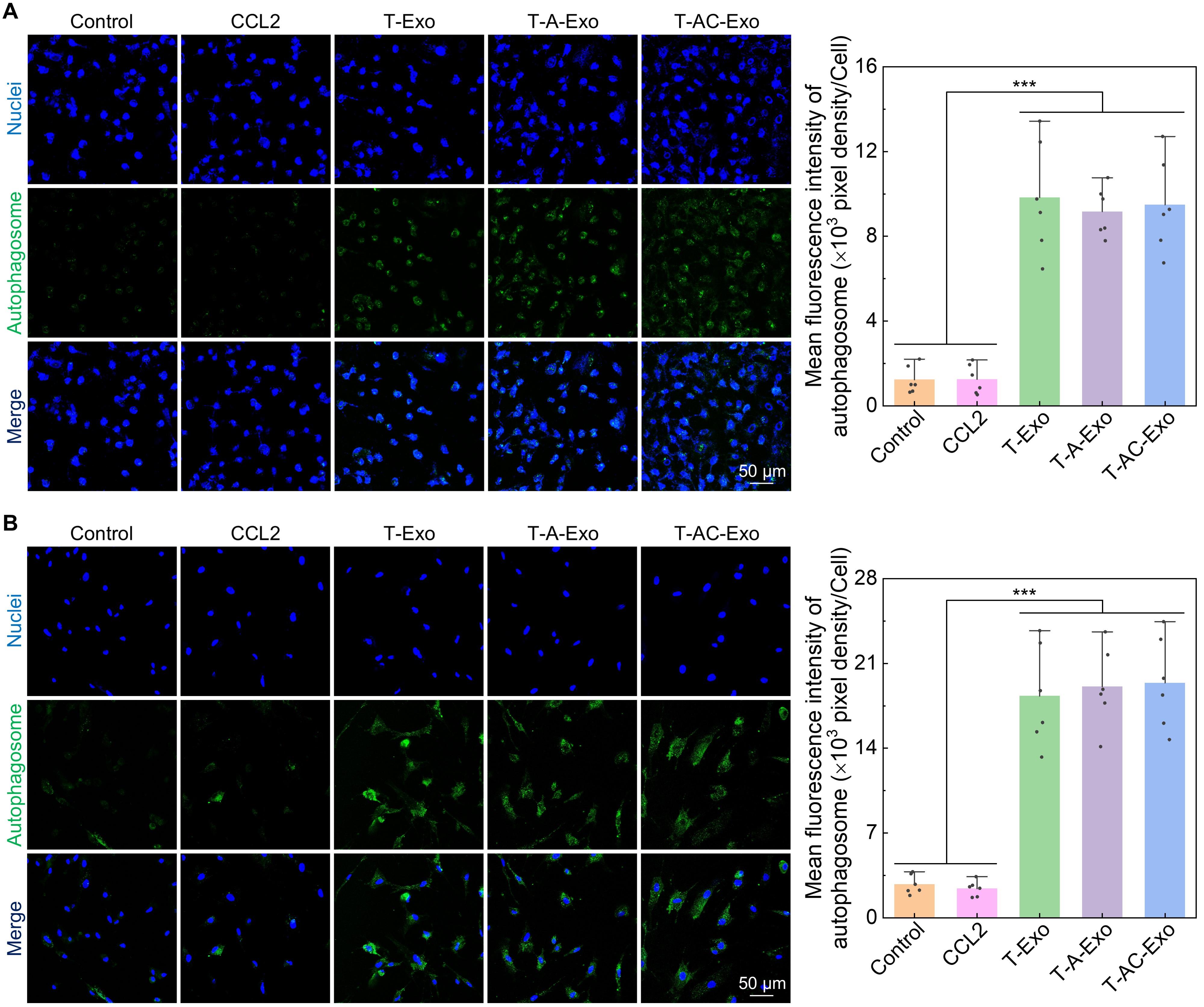


**Figure S13.** Levels of cellular autophagy mediated by CCL2 and MΦ-derived exosomes. A) Fluorescence staining images of autophagosomes and quantification of the mean fluorescence intensity in ECs (n = 6). B) Fluorescence staining images of autophagosomes and quantification of the mean fluorescence intensity in BMSCs (n = 6). Data are presented as means ± SD. ^***^*p* < 0.001.
